# Bipartite Recognition of DNA by TCF/Pangolin Is Remarkably Flexible and Contributes to Transcriptional Responsiveness and Tissue Specificity of Wingless Signaling

**DOI:** 10.1371/journal.pgen.1004591

**Published:** 2014-09-04

**Authors:** Hilary C. Archbold, Chris Broussard, Mikyung V. Chang, Ken M. Cadigan

**Affiliations:** 1 Department of Molecular, Cellular and Developmental Biology, University of Michigan, Ann Arbor, Michigan, United States of America; 2 LSA-IT, University of Michigan, Ann Arbor, Michigan, United States of America; Stanford University School of Medicine, United States of America

## Abstract

The T-cell factor (TCF) family of transcription factors are major mediators of Wnt/β-catenin signaling in metazoans. All TCFs contain a High Mobility Group (HMG) domain that possesses specific DNA binding activity. In addition, many TCFs contain a second DNA binding domain, the C-clamp, which binds to DNA motifs referred to as Helper sites. While HMG and Helper sites are both important for the activation of several Wnt dependent cis-regulatory modules (W-CRMs), the rules of what constitutes a functional HMG-Helper site pair are unknown. In this report, we employed a combination of in vitro binding, reporter gene analysis and bioinformatics to address this question, using the *Drosophila* family member TCF/Pangolin (TCF/Pan) as a model. We found that while there were constraints for the orientation and spacing of HMG-Helper pairs, the presence of a Helper site near a HMG site in any orientation increased binding and transcriptional response, with some orientations displaying tissue-specific patterns. We found that altering an HMG-Helper site pair from a sub-optimal to optimal orientation/spacing dramatically increased the responsiveness of a W-CRM in several fly tissues. In addition, we used the knowledge gained to bioinformatically identify two novel W-CRMs, one that was activated by Wnt/β-catenin signaling in the prothoracic gland, a tissue not previously connected to this pathway. In sum, this work extends the importance of Helper sites in fly W-CRMs and suggests that the type of HMG-Helper pair is a major factor in setting the threshold for Wnt activation and tissue-responsiveness.

## Author Summary

Regulation of gene expression is controlled in large part by proteins known as transcription factors, which bind to specific DNA sequences in the genome. The DNA binding domains of transcription factors recognize short stretches (5–11 base pairs) of DNA with considerable sequence degeneracy. This means that a single DNA binding domain, on its own, cannot find its targets in the vast excess of genomic sequence. We are studying this question using TCF/Pangolin, a *Drosophila* transcription factor that mediates Wnt/β-catenin signaling, an important developmental cell-cell communication pathway. TCF/Pangolin contains two DNA binding domains that bind to a pair of DNA motifs known as HMG and Helper sites. We used a combination of biochemistry, genetics and bioinformatics to elucidate the spacing and orientation constraints of HMG-Helper site pairs. We found that HMG-Helper site spacing/orientation influenced the sensitivity of a target to Wnt signaling, as well as its tissue-responsiveness. We used this information to improve our ability to search the *Drosophila* genome for Wnt targets, one of which was activated by the pathway in the fly ring gland, the major endocrine organ in insects. Our work is relevant to related mammalian TCF family members, which are implicated in development, stem cell biology and the progression of cancer.

## Introduction

During metazoan development, Wnt/β-catenin signaling, often called “canonical” Wnt signaling and hereafter referred to as “Wnt signaling”, is required to drive multiple stage and tissue specific events [1]–[4]. Wnt signaling is essential in such diverse events as specification of the anterior/posterior body axis, and limb, heart, intestinal and craniofacial development [1], [5]–[8]. In several cases, Wnts have been shown to act as morphogens, regulating different targets in a concentration dependent manner [9]–[11]. The pathway is also needed in adult tissues for stem cell maintenance and wound healing [12]–[16], and disregulated Wnt signaling has been implicated in a host of cancers and other human pathologies [17]–[19]. How a single signaling pathway accomplishes such a wide range of outcomes remains a major question in developmental biology and tissue homeostasis.

Variation in Wnt-dependent cis-regulatory modules (W-CRMs) likely contribute to the diversity of Wnt transcriptional responses, though the mechanisms are poorly understood. Members of the T-cell factor (TCF) family of transcription factors (TFs) are principal mediators of Wnt signaling [20], [21]. In many contexts, TCFs act as a transcriptional switch, binding with co-repressors on W-CRM chromatin in the absence of signal, and then recruiting β-catenin and other co-activators in response to Wnt signaling [22], [23]. ChIP-seq studies have found that TCFs co-localize with several other TFs in specific cell types [24]–[31], and combinatorial control may be one method to achieve tissue or temporal specificity. While not as well appreciated, the sequence composition of the TCF binding sites in W-CRMs can also have a major influence on its transcriptional output [32], [33]. A better understanding of the cis-regulatory logic of W-CRMs will shed more light on how they differ in their responsiveness to Wnt signaling, and how TCFs regulate this process.

All TCFs share a highly conserved High Mobility Group (HMG) domain, which binds DNA with sequence specificity [34]–[37]. The HMG recognition motif is a 9–11 bp sequence with the consensus SCTTTGWWSWW. Sequences roughly conforming to this consensus have been shown to be required for activation of numerous W-CRMs [1], [38]. Reporter genes with 3–16 copies of high affinity HMG binding sites behind a basal promoter, such as TOPFLASH, have been used successfully as an experimental readout for Wnt signaling in a number of contexts [38]–[41]. However, such high-density clusters of perfect HMG sites are not found in naturally occurring W-CRMs [1], [38]. Furthermore, there are several instances where synthetic HMG site reporters do not respond to endogenous Wnt signaling in vertebrate tissues [42], [43]. In *Drosophila* embryos and larval imaginal discs, where Wingless (Wg, a fly Wnt) signaling is highly active, synthetic HMG site reporters have little or no expression [38], [44]. These results strongly suggest that under physiological conditions, HMG sites are not sufficient for Wnt activation of W-CRMs.

We have previously reported that several fly W-CRMs contain a GC-rich motif, found near HMG sites, that was critical for Wnt activation [44]. This motif, termed the Helper site, was bound by a second DNA-binding domain in TCF/Pangolin (TCF/Pan, the fly TCF) known as the C-clamp [44]. The C-clamp was originally discovered in “E-tail” isoforms of mammalian TCF1 and TCF4 genes [45]. These TCF isoforms also bound Helper sites, which were essential for the activation of specific mammalian W-CRMs [45]–[47]. Reporters containing only multimerized copies of Helper sites did not respond to Wnt signaling, but these motifs synergized with HMG sites to greatly enhance the Wnt activation of reporter constructs [44]. The presence of an intact C-clamp domain imparts increased affinity for DNA containing both HMG and Helper sites and a functional C-clamp is required for TCF/Pan activation of fly W-CRMs [44], [48]. These data support a bipartite binding model for C-clamp containing TCF family members, where HMG domain-HMG site and C-clamp-Helper site interactions allow TCF to properly locate W-CRMs and regulate Wnt target genes.

**Figure 1:**
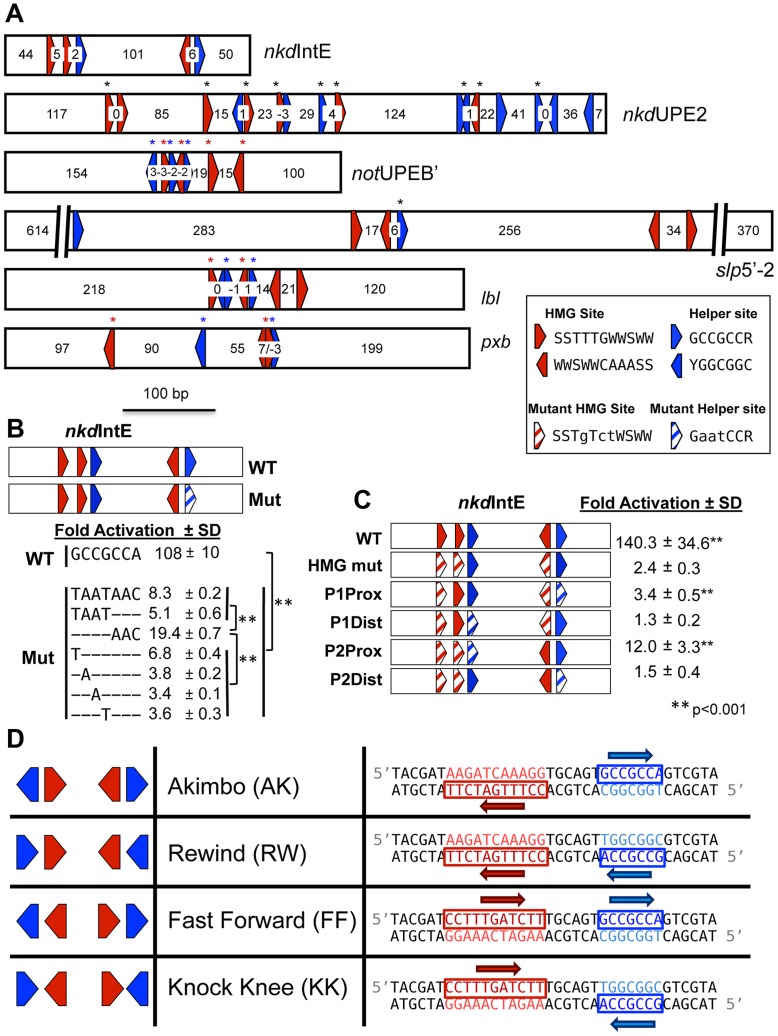
HMG and Helper site configurations in W-CRMs. (A) Previously characterized W-CRMs [44], [57] with location of predicted HMG (red arrows) and Helper (blue arrows) indicated (see Figure S3 for PWMs of these motifs). Cutoffs for HMG and Helper sites were 5.35 and 6.5, respectively. Direction of arrow indicates orientation of motif (see inset for consensus sequences in both orientations). Number of nucleotides between each motif is indicated. Black asterisks indicate sites that contributed to W-CRM activation by Wnt signaling in cell culture when mutated individually [44], [57]. Red and blue asterisks denote function when all indicated HMG or Helper sites were mutated simultaneously. (B) Systematic mutagenesis of second Helper site in the *nkd*IntE W-CRM reporter indicates all seven positions contribute to W-CRM activation. Letters indicate mutated nucleotides, with dashes denoting the wild-type sequence. Reporter constructs were transfected into *Drosophila* Kc cells with or without a plasmid expressing Arm*. Fold activation represents the ratio of Arm*/control, SD equals standard deviation of three biological replicates. (C) HMG and Helper sites work in closely spaced pairs. The *nkd*IntE reporter contains three functional HMG and two functional Helper sites [44], which were mutated (striped arrows) in different combinations, and tested for Arm* activation in Kc cells. Values represent the mean of three biological replicates ± SD. A Student's T-test was employed to determine significance for data in panels B and C. (D) Nomenclature and symbology for the four possible HMG-Helper site pair orientations. A right pointing arrow indicates the consensus sequence is read 5′ to 3′ on the “top” strand, a left arrow indicates the consensus is read 5′ to 3′ on the “bottom” strand. The sequences shown for HMG and Helper sites are identical to the ones used for DNA binding experiments in Figure 2 and synthetic Wnt reporter constructs (Figure 3A, 4 and 5).

Surprisingly, our initial characterization of Helper site sequences in *Drosophila* W-CRMs identified numerous putative Helper elements with variable spacing and orientation with respect to HMG sites (Figure 1A). This was interesting, because bipartite binding by TFs is typically very sensitive to the spacing and orientation of the two sites. Examples of this spacing/orientation constraint include several type II nuclear receptor/RXR heterodimers [49]–[51] and Smad heterodimers [52], [53]. Spacing and orientation is also important for the POU family member Pit-1 [54], [55], and the spacing of half-sites has been shown to determine whether target genes are activated or repressed. In contrast, the related zinc finger DNA binding proteins SIP1 and δEF1 have a high tolerance for half-site spacing and orientation variability, perhaps because the two DNA-binding zinc finger clusters are separated by a large and presumably flexible linker region [56]. Given the short (10 aa) spacer between the HMG and C-clamp domains in TCF/Pan, it was unclear whether all the variable HMG-Helper site pairs found in W-CRMs were *bona fide* TCF binding sites. As no consistent organizational preference was seen between the functional HMG and Helper sites (asterisks, Figure 1A), a systematic approach was needed to determine the constraints of HMG-Helper pair flexibility.

In this report, we examined the rules of TCF/Pan binding to HMG-Helper site pairs using several experimental approaches. We identified two HMG-Helper site configurations that were bound by TCF/Pan with highest affinity in vitro, one where the Helper site is located 6 bp upstream of the HMG site, and the other where it is immediately adjacent downstream. These two HMG-Helper site configurations also had the greatest transcriptional activity in many tissues, and were most enriched in genomic regions bound by TCF/Pan. We suggest a model where the DNA-bending activity of the HMG domain enables TCF/Pan to recognize both these HMG-Helper site configurations. However, our data also make clear that the presence of a Helper site near a HMG site in any orientation and with variable spacing enhanced TCF/Pan binding, and many of these “non-optimal” arrangements had transcriptional activity, some with striking tissue-specificity. In addition, we have shown that altering the orientation/spacing of an HMG-Helper site pair in a W-CRM has a dramatic effect on its sensitivity to the Wg morphogen in imaginal tissues. Finally, we used our knowledge of the cis-regulatory code for TCF/Pan binding to informatically identify new W-CRMs. One of these drove expression in the prothoracic gland (PG), a major component of larval ring gland, an endocrine tissue not previously linked to Wg signaling. We found that Wg is expressed in the ring gland, and that blocking Wg signaling in this organ resulted in early larval developmental arrest. These findings highlight how a better understanding of DNA recognition by TCF/Pan can enhance our ability to identify novel W-CRMs and discover new aspects of Wnt biology.

## Results

### HMG and Helper Sites Work in Pairs

The *Drosophila* Helper site was previously defined by sequence alignment of several functional motifs as having the consensus GCCGCCR (R = A/G) [44]. However, a shorter consensus has been reported for vertebrate E-tail TCFs (RCCG) [45]. To test whether all seven nucleotides of the longer consensus were required for maximal activation, we performed serial mutagenesis on the second Helper motif in the *nkd*IntE W-CRM luciferase reporter (Figure 1B). This reporter was highly activated by expression of Armadillo (Arm, the fly β-catenin), which contains a point mutation rendering it resistant to degradation (Arm*) [44], [57]. Substitution of any of the first four positions had as dramatic a reduction in reporter activation as mutating the entire 7 bp motif. Mutation of the last three positions had a slightly less severe reduction (Figure 1B). Thus, at least in this context, all seven bp in the GCCGCCR motif are important for maximal activation by Wnt signaling.

Previous evidence supported the idea that HMG and Helper sites work in closely spaced pairs. For example, the contribution of individual HMG sites to W-CRM activation varied widely, with HMG sites proximal to Helpers sites more likely to contribute to activation [44], [57]. To further test the HMG-Helper site pair hypothesis, we again used the *nkd*IntE W-CRM, previously found to contain three functional HMG binding sites, and two functional Helper sites [44]. The arrangement of these functional sites suggests that there are two closely spaced HMG-Helper site pairs, separated by 101 bp (Figure 1A), but there remained a formal possibility of longer-range interactions between HMG and Helper sites.

As previously reported [44], activation by Arm* is nearly abolished by mutation of the three HMG binding sites (Figure 1C). Four additional *nkd*IntE mutants were created, leaving one HMG and one Helper site intact. The two constructs retaining a HMG site and Helper site in close proximity activated target gene transcription at levels higher than the HMG mutant control. The first pair had a small but reproducible activation, while the activation of the second intact pair was more pronounced (Figure 1C). In contrast, the reporters where the intact HMG and Helper sites were separated (P1Dist & P2Dist) were not activated. These data support the idea that HMG and Helper sites must be in close proximity to respond to Wnt signaling.

### TCF/Pan Prefers Specific HMG-Helper Configurations *In Vitro*

There are four possible orientations for HMG-Helper site pairs, which we have termed Akimbo (AK), Rewind (RW), Fast Forward (FF), and Knock Knee (KK) (Figure 1D). Helper sites are defined by the aforementioned seven bp GCCGCCR consensus (Figure 1B). We used the eleven bp consensus of SCTTTGWSWW determined for TCF/Pan [58] to define HMG sites. It should be noted that the four orientations indicate the relationship between the HMG and Helper sites, and not the relationship of these bipartite motifs to the nearest transcription start site (TSS). Therefore, it is possible to have either the Helper or HMG site first in all four orientations, depending on which strand contains the consensus (Figure 1D). The spacing of each pair is defined by the number of bp between the two motifs, e.g., the examples in Figure 1D have a spacing of 6 bp and will hereafter be referred to as AK6, FF6, etc.

**Figure 2:**
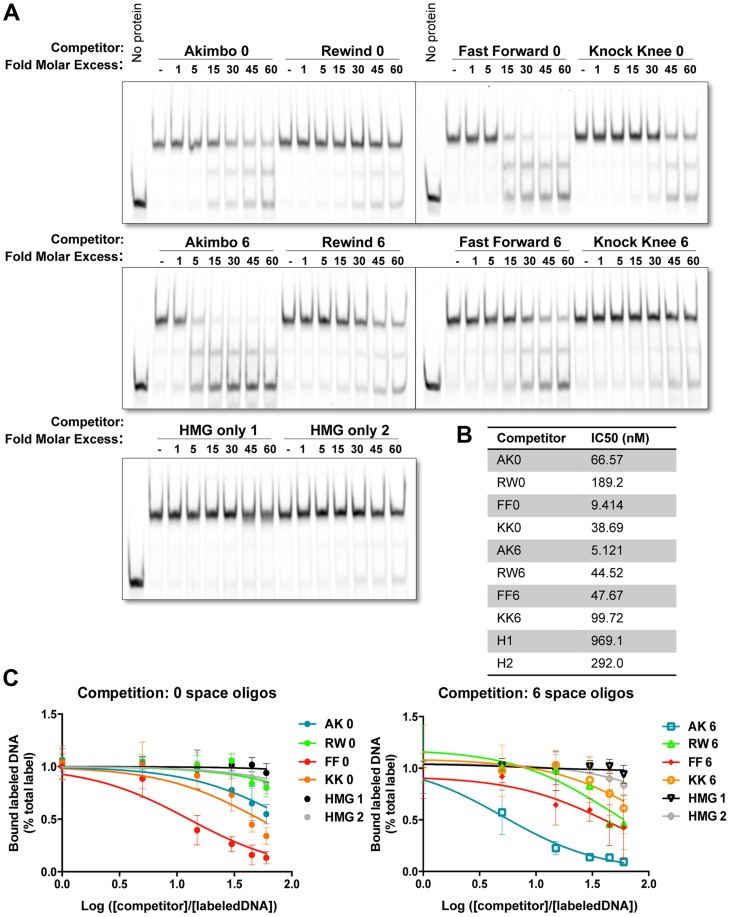
Binding preferences of TCF/Pan for various HMG-Helper site configurations *in vitro*. (A) Competition electromobility shift assay (EMSA) experiments performed with a recombinant TCF fragment containing the HMG and C-Clamp domains, an AK6 IR-labeled probe, and competitor oligonucleotides containing one of the four orientations at 0 or 6 spaces. Images were taken on the Licor Odyssey, and binding intensity quantified with Image Studio 2.0. AK6 and FF0 were the strongest competitors, but Helper sites in all positions improve binding affinity when compared to binding of the HMG sites alone (HMG only 1 and 2). (B) The IC_50_ value (the measure of DNA concentration required to reduce binding of the labeled probe to 50% of uncompeted levels) for each competitor was calculated using Prism 6.1 (Graphpad). (C) Semilog graphs depicting competition results from three independent experiments. Error bars indicate SD. Sequences of the HMG and Helper motifs the same as shown in Figure 1D (see Table S1 for full sequence of each competitor).

We previously reported that the presence of a Helper site increased the ability of TCF/Pan to bind to DNA in vitro [44]. These experiments utilized an AK5 HMG-Helper site configuration. To determine the relative binding affinities of different HMG-Helper site pairs, we performed electromobility shift assays (EMSAs) with a recombinant His-tagged protein containing both the HMG and C-Clamp domains of TCF/Pan, a labeled AK6 probe (see Figure 1D for sequence) and unlabeled competitor oligonucleotides containing the 0 and 6 bp versions of each orientation. The AK6 probe was labeled with an infrared (IR)-dye, allowing quantification of the gel shift with the Licor Odyssey IR platform (see Materials and Methods for further details). Representative blots are presented (Figure 2A) and the data from multiple experiments are summarized by showing the half maximal inhibitory concentrations (IC_50_) for each competitor (Figure 2B) and the dose-response curves on semi-log line graphs (Figure 2C).

The competition assays clearly showed that TCF/Pan had a preference for oligonucleotides containing an AK6 or FF0 motif. The IC_50_ for AK6 and FF0 were 5.1 and 9.4 nM, respectively (Figure 2B). RW6, KK0, FF6 and AK0 were in the next group, with IC_50_'s between 38.7–66.6 nM. KK6 and RW0 had the lowest relative affinity (IC_50_ of 99.7 and 189 nM, respectively), which was still greater than two HMG site only oligonucleotides (IC_50_ of 292 and 969 nM) (Figure 2B). The data indicate that AK6 and FF0 are bound with the greatest affinity by TCF/Pan, but also demonstrate that the presence of a nearby Helper site in any orientation enhances its recognition by TCF/Pan.

### HMG-Helper Site Configuration Preferences in Cell Culture Assays

**Figure 3:**
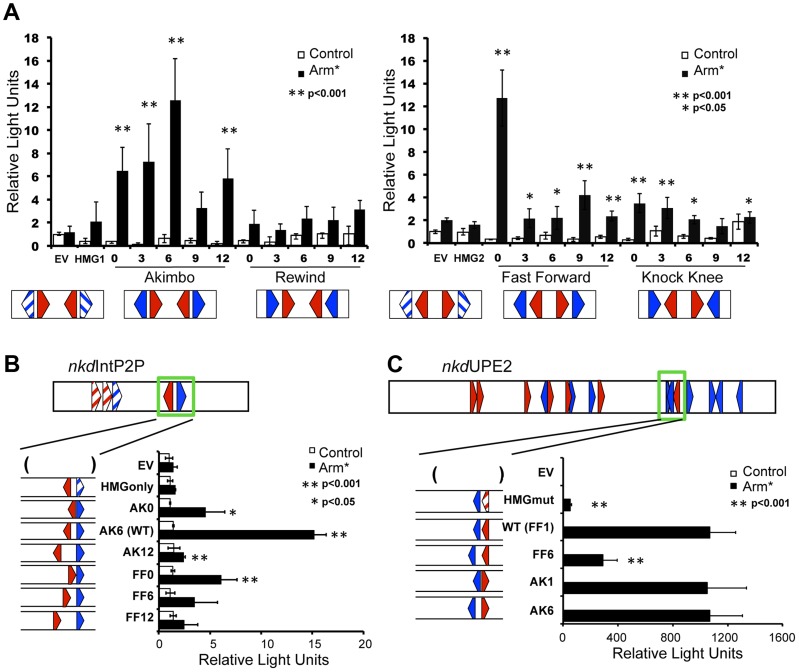
HMG-Helper pair configuration preferences in cell culture reporter assays. Kc cells were transfected with the indicated reporters with or without a plasmid expressing Arm* and assayed for luciferase expression. (A) Synthetic W-CRMs containing two HMG-Helper site pairs in all four orientations with 0, 3, 6, 9 or 12 bp spacing between the HMG and Helper site (see Table S1 for sequences of each synthetic W-CRM). In every construct, the two pairs were separated by 6 bp. Two constructs containing only HMG sites (HMG1 and HMG2) were included in the analysis and the reporter vector without an insert is referred to as empty vector (EV). The data shown represents the average of three independent experiments with three biological replicates each. Error bars represent SD and asterisks indicate a significant difference in activation compared to HMG site constructs. (B) The cartoon at the top of the panel depicts the *nkd*IntE2P2 W-CRM, all HMG and Helpers sites were mutated (striped arrowheads) except one AK6 pair (green box). The spacing and orientation of this pair were altered as indicated. The endogenous AK6 configuration displayed the highest level of activation by Arm*, while AK0, AK12 and FF0 constructs also exhibited higher activation than the HMG site only control. The data are the means of two independent experiments with three biological replicates each, ± SD. Asterisks indicate a significant increase in activation compared to the HMG site only construct. (C) In the *nkd*UPE2 W-CRM, the HMG and Helper site pair (green box) which contributes most potently to activation [57] was altered as indicated. AK1 and AK6 configurations responded as well as the endogenous FF1 pair, while the FF6 configuration was less active. The data are the means of three biological replicates ± SD, and are representative of several independent experiments. Asterisks indicate reduced activation by Arm* compared to the wild-type construct. In all cases, Student's T-tests were employed to determine significance.

To explore the functional orientation/spacing constraints between various HMG-Helper site configurations, we created a series of synthetic W-CRMs containing two HMG-Helper site pairs upstream of a minimal promoter. All four orientations were tested for the ability to activate a luciferase reporter gene at 0, 3, 6, 9 and 12 bp spacing in transfected Kc cells (see Supplemental S1 for complete sequences used). Three out of the four orientations (AK, FF & KK) exhibited levels of activation by Arm* higher than reporters containing HMG sites alone or the empty vector (EV; Figure 3A). Spacing of HMG-Helper pairs affected the level of activation in an orientation-dependent manner. The AK reporters were significantly different from the HMG site only reporters at most spacings tested, but peak activity occurred with AK6 (Figure 3A). In contrast, for FF, activation was greatest at 0 bp spacing, with much weaker activation at greater distances. The KK orientation constructs showed weak activation at several spacings, though activation was slightly greater when the HMG and Helper sites were closer together. In contrast to the other three orientations, the RW reporters were not able to activate gene transcription more potently than the HMG site controls at any of the spacings tested (Figure 3A).

To explore the spacing requirements of the AK and FF HMG-Helper site pairs in the context of endogenous enhancers, we chose two previously characterized W-CRMs from the *nkd* locus. First, we used a modified *nkd*IntE, termed *nkd*IntEP2P, where the first two HMG sites and Helper are mutated, leaving only the endogenous AK6 motif (Figure 3B). We replaced this motif with either AK or FF motifs containing 0, 6, or 12 bp spacers. In this context, AK6 promoted the most robust activation, while the AK0 and AK12 constructs had lower levels of activation, consistent with the behavior of the synthetic constructs. Also consistent with the synthetic data, FF0 was the only spacing of the FF *nkd*IntEP2P constructs to activate at levels significantly different than the HMG only control (Figure 3B).

We then examined a second W-CRM, *nkd*UPE2, previously shown to have a specific HMG and Helper site that were major contributors to Wg activation [57]. This HMG-Helper pair (green box in Figure 3C cartoon) has a degenerate FF1/KK0 conformation. Mutation of the HMG site resulted in a dramatic decrease in activation by Arm* (Figure 3C). We altered this HMG-Helper site pair to an AK1, AK6, or FF6 configuration. The AK motifs were more flexible in the range of functional spacing, as both AK1 and AK6 containing W-CRMs activated transcription as robustly as the WT FF1 element (Figure 3C). The FF motif displayed a strong preference for the 1 bp spacer configuration, with strongly decreased activation from the FF6 element (Figure 3C). However, the FF6 motif retained some activation by Arm*, as compared to the HMG site mutant (Figure 3C). The data with the *nkd*IntE and *nkd*UpE reporters indicate that the configurations that worked well (e.g., AK6, FF0) in the synthetic reporters in cell culture (Figure 3A) also are optimal for the *nkd* W-CRMs in cell culture reporter assays. It should also be noted that increasing the spacing of the HMG-Helper site pairs (e.g., AK12, FF6-12) always resulted in a decrease in transcriptional activity, consistent with a requirement for these motifs to be relatively near each other.

### HMG-Helper Site Synthetic Reporters Reveal Tissue-Specific Expression in *Drosophila* Tissues

**Figure 4:**
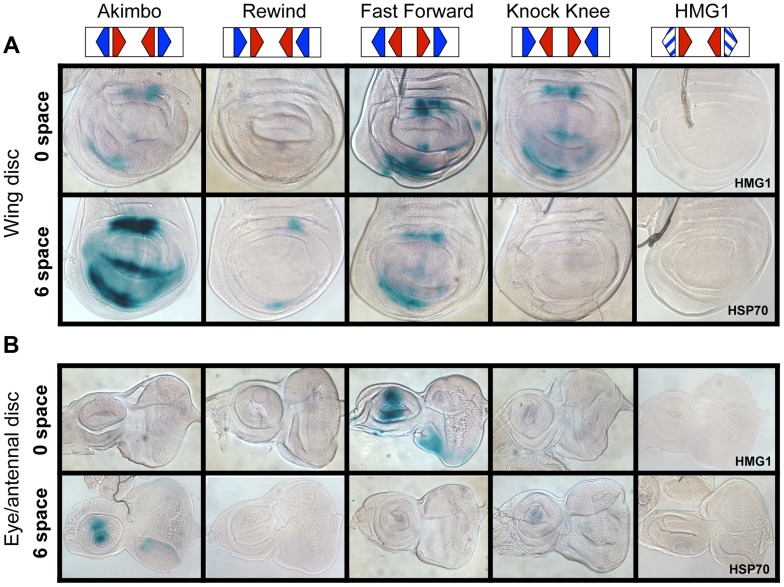
HMG-Helper pair configuration preferences in imaginal discs. Brightfield images of imaginal discs from 3^rd^ instar larva containing the indicated lacZ reporter constructs stained for lacZ activity. (A) Wing imaginal discs. (B) Eye/antennal discs. The FF0 and AK6 reporters display the highest expression in these tissues. Neither the promoter alone (HSP70) nor the HMG site only (HMG1 shown) constructs have detectable expression. At least 20 discs for each reporter were analyzed, with representative images shown. The same base pair sequences used in Figure 3 were utilized for the transgenic reporters (see Table S1 for sequences).

To test whether the functional constraints for HMG-Helper site configurations observed in cell culture assays also held true in the context of an intact organism, transgenic reporter lines with different HMG-Helper pairs were generated in *Drosophila*. ΦC31 site directed integration of reporter constructs was utilized to eliminate position effects [59]. All four orientations at 0 and 6 spaces were tested, as these HMG-Helper pairs displayed distinct outputs in cell culture (Figure 3A). The same sequences used in the cell culture reporters were utilized in the transgenic reporters (sequences provided in Table S1). None of the constructs displayed strong expression during embryogenesis (Figure S1). In contrast, in imaginal discs from wandering 3^rd^ instar larva, several HMG-Helper site reporters had expression patterns consistent with activation by Wg signaling (Figure 4A, 4B) [1], [60]–[62]. These activities were similar to the expression pattern of Wg (Figure S2), and were Helper site dependent, as the HMG site only reporters had no detectable expression over the basal *Hsp70* promoter (Figure 4A, 4B). Consistent with our cell culture data, the most potent activity was seen with both the AK6 and FF0 HMG-Helper pairs (Figure 4A, 4B). Other configurations (AK0, FF6, KK0) displayed weaker expression. Interestingly, with the exception of RW0, the presence of Helper sites in all other orientation/spacings tested displayed more activity than the HMG site only controls in the imaginal discs (Figure 4A, 4B). These results indicate that Helper sites have a surprising degree of flexibility in potentiating the ability of HMG site to respond to Wg signaling.

**Figure 5:**
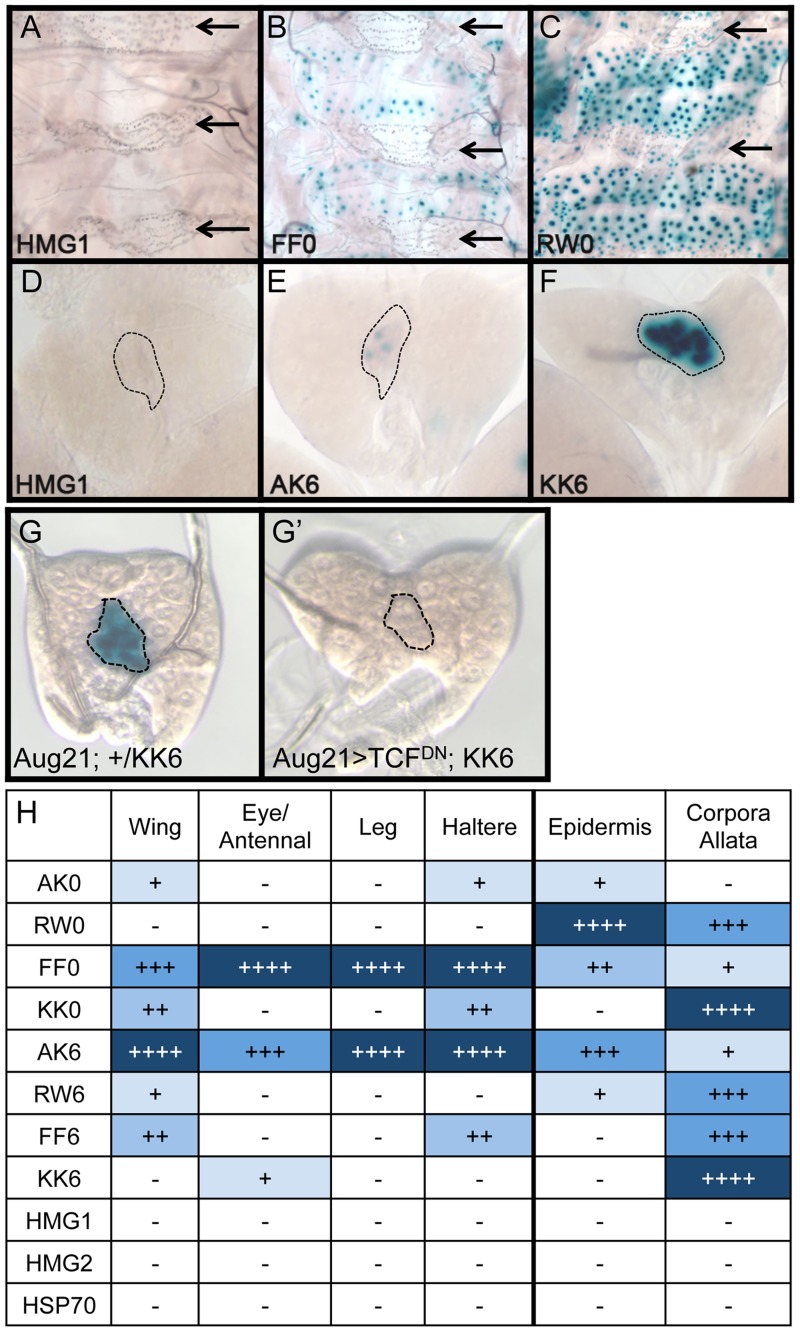
Tissue-specific activity of HMG-Helper pair reporters. Brightfield images of tissues from 3^rd^ instar larva containing the indicated lacZ reporter constructs stained for lacZ activity. (A–C) LacZ expression in larval epidermis. Expression is seen in cells underlying the naked cuticle, located between denticle belts (arrows). The HMG1 reporter has no detectable expression; FF0 has weak expression and RW0 drives robust expression. (D–F) Ring glands with expression in the Corpora Allata (dotted lines). HMG1 has no detectable expression with AK6 and KK6 displaying weak and strong expression, respectively. (G–G′) Expression of TCF^DN^ in the CA (via the Aug21Gal4 driver) abolishes expression of the KK6 reporter. (H) Summary of expression data from all tissues examined, with number of plus signs and blue hue indicative of the relative level of reporter gene expression. At least 12 samples of each reporter line were analyzed for each tissue, with similar results observed between individual samples.

While the AK6 and FF0 synthetic reporters displayed the most activity in imaginal discs, there were tissue-specific differences in their expression. AK6 was the most robust responder to Wg signaling in wing imaginal discs (Figure 4A), while FF0 was the most highly expressed reporter in eye/antennal discs (Figure 4B). In some non-imaginal tissues, the other two orientations displayed the highest level of activation. For example, RW0 drove robust expression in the larval epidermis, in the cells underlying the naked cuticle located between denticle belts (Figure 5C), while other generally favorable configurations, like FF0, had less expression (Figure 5B). In addition, the AK6 reporter had extremely weak expression in the corpora allata (CA), also known as the medial secretory cells of the ring gland (Figure 5E), while KK6 was expressed at much higher levels (Figure 5F). This expression was completely inhibited by expression of a dominant negative version of TCF/Pan (TCF^DN^) [58] in the CA (Figure 5G′).

A summary of all the collected expression data from the eight HMG-Helper site reporters is shown in Figure 5H. FF0 and AK6 were clearly the strongest reporters in imaginal discs and had intermediate expression in the epidermis. However, they were weakly expressed in the CA. Strikingly, RW0, which had no detectable expression in the imaginal discs, displayed high expression in the epidermis and CA. KK0 & KK6 had weak expression in the discs, no activity in the epidermis and the highest expression in the CA (Figure 5H). These data suggest the possibility that altering HMG-Helper site architecture may be a way to create a repertoire of tissue-specific responses to Wg signaling.

### TCF/Pan-Bound Embryonic Chromatin Is Enriched for Optimal HMG-Helper Site Configurations

The in vitro DNA binding assays described earlier (Figure 2) are a reductionist approach to understanding HMG-Helper site recognition by TCF/Pan. An alternative is to determine whether HMG-Helper site pairs are enriched in genomic sequences bound by TCF/Pan. A genome-wide survey of TCF/Pan localization in germband extended *Drosophila* embryos was performed and made publicly available [28]. Germband extension is a developmental stage when Wg signaling is patterning the embryonic epidermis and mesoderm [63]–[66]. For one timepoint (6–8 hr after fertilization), 2079 high confidence TCF/Pan peaks were identified [28]. We analyzed the DNA covered by these TCF/Pan peaks (∼2.9×10^6^ bp) for HMG-Helper pairs and compared these regions to equivalent randomly selected intronic and intergenic DNA.

To analyze these genomic sequences, we created a program to identify HMG and Helper site pairs, which could then be sorted for orientation and distance (see Materials and Methods). Position Weight Matrices (PWMs) of each motif were created from the collection of functional HMG and Helper sites we have identified [44], [57](Figure S3). This allowed us to analyze DNA sequences using different stringencies for calling HMG and Helper sites. We considered PWM values of 4.5 for HMG sites and 6.5 for Helper sites to be a fairly stringent criteria for these motifs, while 3.5 and 5.0 (for HMG and Helper sites respectively) was considered a more relaxed calling criteria.

**Figure 6:**
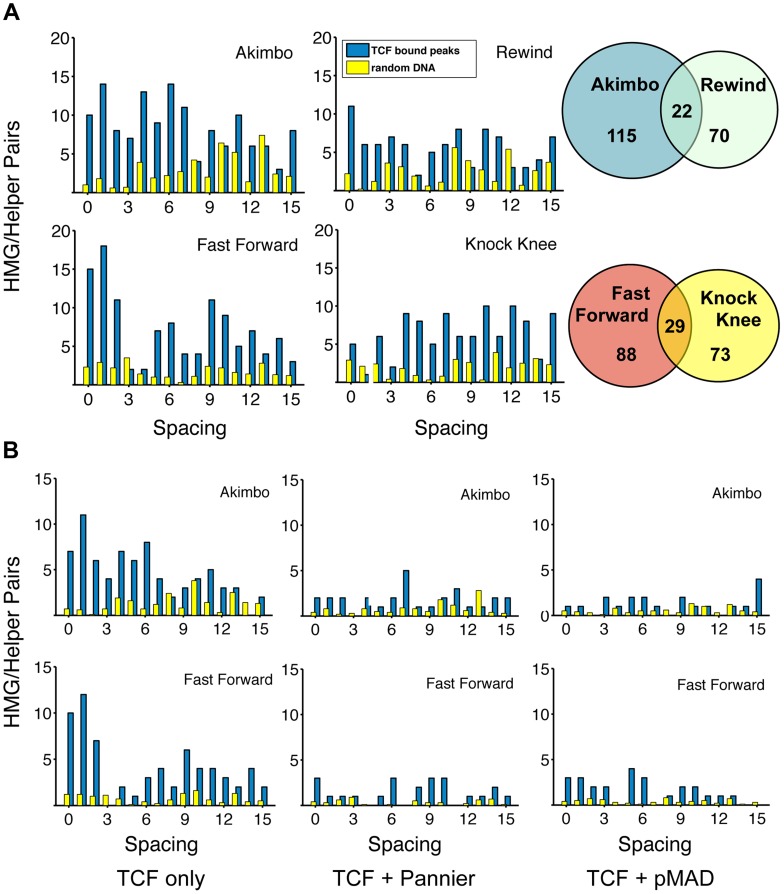
TCF/Pan-bound chromatin is enriched for HMG-Helper site pairs. (A) Distribution of HMG-Helper pairs in genomic sequences bound by TCF/Pan (blue bars) versus random DNA (yellow bars). TCF/Pan-bound sequences were obtained from a ChIP-seq data set from germband extended *Drosophila* embryos [28]. Equivalent amounts of random DNA from intergenic, intronic and 5′/3′UTR regions were analyzed, with the average of ten such runs displayed. Nearly all the HMG-Helper site configurations were enriched in TCF/Pan bound regions, with the highest degree of enrichment in FF0-2 and AK0-6. Due to the semi-palindromic nature of the Helper site, many sites with nucleotide mismatches were called as both AK/RW or FF/KK. The overlap is indicated in the Venn diagram to the right of each pair of graphs. (B) AK and FF motifs are highly enriched in peaks which are uniquely bound by TCF/Pan (see text for further explanation). Enrichment is less dramatic in TCF/Pan peaks that are shared by Pannier or pMAD.

Regardless of the criteria used, HMG-Helper pairs were enriched in the TCF/Pan bound regions. With the stringent criteria, pairs with 0–15 bp spacers were 3.48 times more likely to occur in bound peaks than in random DNA (Figure 6A). This enrichment level was considerably higher than that obtained for HMG sites only (1.46 times enriched in bound DNA) or for the Helper sites, which were underrepresented in bound DNA (0.76 times) compared to random DNA. Using the relaxed criteria for calling motifs, many more HMG-Helper sites were identified (2139 versus 448), and they were 2.4 fold enriched in TCF/Pan bound versus random DNA (see Figure S4).

A closer look at the spacing between HMG-Helper pairs in all four orientations revealed two general messages. First, the enrichment over random DNA was most pronounced in configurations that were favorable for in vitro binding and/or transcriptional activity in cell culture and imaginal discs. For example, at the stringent calling criteria, FF0-2 and AK0-6 pairs were 6.1 times as likely to be found in TCF/Pan bound compared to random DNA (Figure 6A). Second, despite this first point, it was also true that HMG-Helper sites in every orientation at almost every spacing were enriched in TCF/Pan bound DNA (Figure 6A), and this was also true at the more relaxed criteria for calling motifs (Figure S4). It should also be noted that there were a number of palindromic motifs (e.g. YGCCGGCR) that were double called, either as both AK and RW or as both FF and KK. These pairs are represented as the overlapping area in the Venn diagrams (Figure 6A).

In addition to examining TCF/Pan localization in the *Drosophila* genome, Junion and co-workers surveyed four other TFs involved in cardiogenesis: the GATA factor Pannier, phosphorylated Mad (pMAD), Tinman (Tin) and Dorsocross (Doc). They found that many genomic locations contained several of these TFs, which often contained functional W-CRMs that were active in cardiac or mesodermal cells [28]. To determine if the frequency of HMG-Helper site pairs was different at sites where TCF/Pan co-localized with these TFs, we partitioned the TCF/Pan bound peaks into those in which the peak center was within 150 bp of another TF's peak, and those in which the center was not within 150 bp of any of the tested TFs. We called this latter class of peaks “TCF unique”, though this is only known for the TFs included in the analysis. This caveat aside, it is still interesting to note that FF0-2 and AK0-6 pairs were 16.25 times more likely to be found in the TCF unique peaks compared to random DNA, while these motifs were less enriched in the peaks shared with Pannier (4.42 fold) and pMad (3.78 fold) (Figure 6B; Figure S5). Even less enrichment was observed in the peaks TCF/Pan shared with Tinman and Dorsocross (3.07 & 1.80 fold, respectively) (Figure S5). These data suggest that the mechanism(s) for recruitment of TCF/Pan to chromatin differs depending on the prevalence of co-localizing TFs.

### Altering HMG-Helper Site Architecture Increases W-CRM Sensitivity to Wg Signaling

**Figure 7:**
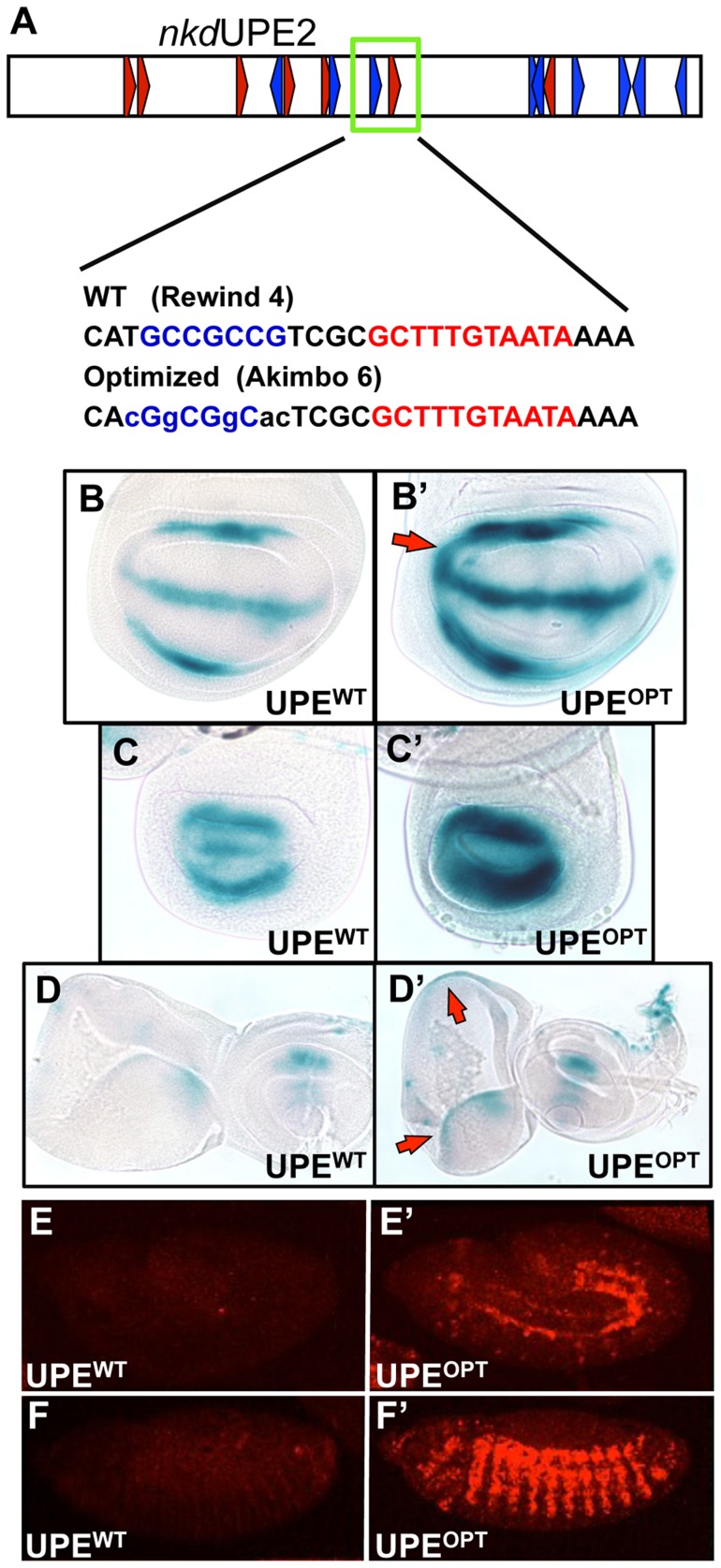
Optimizing motif architecture increases transcriptional output. (A) Cartoon of the *nkd*UPE2 W-CRM. A RW4 motif (green box) was altered to an AK6, point mutations indicated in lowercase. (B–D) Brightfield images of 3^rd^ instar imaginal discs stained with X-gal. The “optimized” AK6 containing (OPT) W-CRM (B′, C′, D′) drives higher expression levels of the reporter transgene than the wildtype (WT) W-CRM (B,C,D), and expands the region which responds to Wg signaling (red arrows). Representative wing (B), haltere (C), and eye/antennal (D) imaginal discs shown. (E,F) Confocal images of lacZ immunostained *nkd*UPE2 W-CRM reporters at embryonic stage 12 (E, E′) and stage 13 (F, F′). The “optimized” reporter has a dramatic increase in expression.

We next wanted to test if we could alter the activity of an endogenous W-CRM in vivo by replacing a suboptimal HMG-Helper site pair with an “optimal” configuration. *nkd*UPE2 was a good candidate, since this W-CRM is active in the imaginal discs [44], [57], and contains an endogenous RW4 HMG-Helper site pair (green box, Figure 7A) which contributes only weakly to activation by Wg signaling in cell culture [57]. The RW4 motif was reconfigured to an AK6 pair through site-directed mutagenesis (Figure 7A). Strikingly, this “optimized” W-CRM reporter displayed increased expression in the wing, haltere and eye/antennal imaginal discs, as well as in the embryonic epidermis (Figure 7B–7F′). The domain of reporter gene expression was also increased in the wing discs (arrows in Figure 7B, 7B′). The expression of the optimized reporter was inhibited by TCF^DN^ (Figure S6), as we have described previously for the wild type reporter [57]. These results suggest that the optimized W-CRM has greater sensitivity to the secreted Wg morphogen.

### *In Silico* Searches for Novel W-CRMs Using Optimized HMG-Helper Site Architecture

Previously, we used in silico searches for clusters of HMG and Helper sites to identify novel W-CRMs, without factoring in the orientation and spacing of potential HMG and Helper site pairs [44]. As our data indicate certain conformations, such as FF0-1, are overrepresented in TCF/Pan-bound DNA and drive robust activation by Wg signaling in multiple contexts, we tailored a computational search for FF1 motifs. A stringent calling criterion was used, to keep the number of hits at a manageable level. The search was performed on the right arm of chromosome 3, containing more than 20 Mb of sequence, using Target Explorer, an on-line search algorithm [67]. The stringent criteria resulted in a short list of 23 hits (Figure S7). We chose two putative W-CRMs that contained additional lower stringency HMG-Helper pairs near the initial FF1 hit for further analysis.

**Figure 8:**
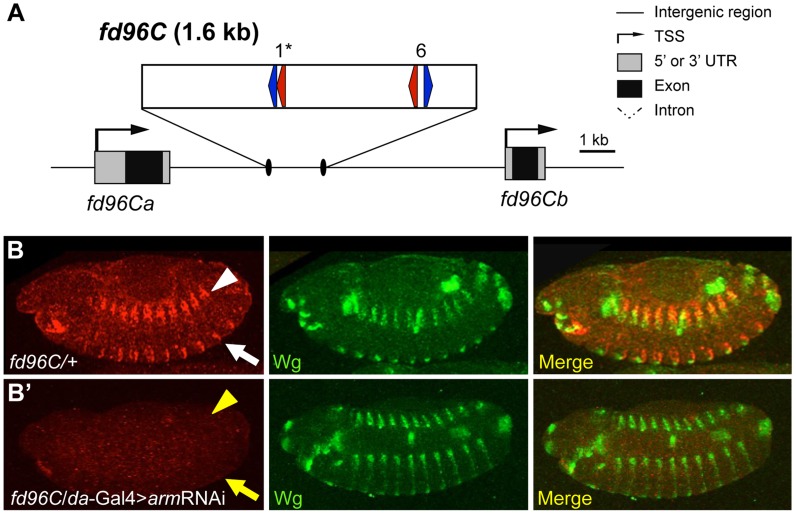
Computationally identified W-CRM in the *fd96C* locus. (A) Cartoon of the *fd96C* region, displaying the location of the W-CRM between *fd96Ca* and *b* genes (UTRs in gray, coding regions in black). The position of HMG (red arrows) and Helper (blue arrows) sites and their spacing in the W-CRM is indicated. Asterisks indicate the high scoring FF1 motif identified in the initial computational search. (B) The *fd96c* W-CRM drives expression in both the dorsal (white arrowhead) and ventral (white arrow) stripes overlapping the Wg expression domain. (B′) RNAi depletion of Arm results in loss of reporter gene activation (yellow arrow head and arrow).

One W-CRM is located in the intergenic region between the related genes *forkhead domain containing 96C a* and *b* (*fd96Ca* and *fd96Cb*) (Figure 8A). A transgene containing this W-CRM driving lacZ was robustly expressed in ventral and dorsal stripes after germband retraction, in a pattern overlapping the expression of Wg (Figure 8B). To confirm that the reporter was dependent on Wg signaling, we examined its expression in embryos where Arm was depleted by driving an *arm*RNAi transgene via the ubiquitous *daughterless* (*da*)-Gal4 driver [68]. Arm depletion resulted in a nearly complete loss of reporter expression (Figure 8B′). In addition to its role in Wg signaling, Arm is also required for cell adhesion [69], [70], raising the possibility that depletion of Arm indirectly effects expression of the W-CRM reporter. This is unlikely, because *da*Gal4>UAS*arm*RNAi embryos were morphologically normal at stage 13 and had normal expression of Wg (Figure 8, 9B′). In addition, these embryos secreted cuticle with the standard patterning defects seen with reduced Wg signaling [63], [71], but no cuticle defects associated with loss of cellular adhesion [69], [70]; (Figure S8). These data indicate that the cis-regulatory element identified between *fd96Ca* and *fd96Cb* is a bona fide W-CRM.

**Figure 9:**
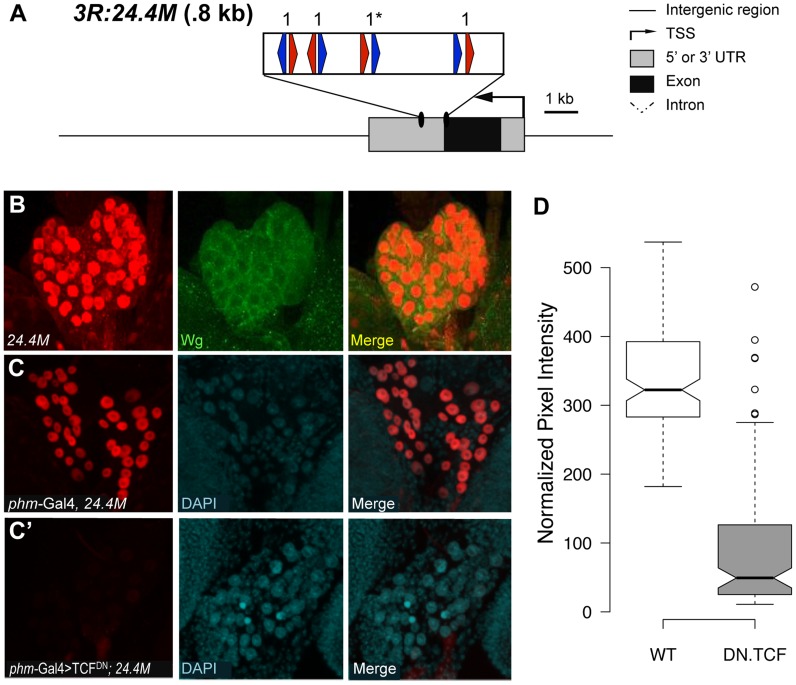
Identification of a W-CRM at 3R:24.4M that is active in the ring gland. (A) Cartoon of the W-CRM in relation to the *fkh* gene, with the location of HMG (red arrows) and Helper (blue arrows) sites indicated, along with their spacing. Asterisks indicate the high scoring FF1 motif identified in the initial computational search. (B) Confocal images of a 3^rd^ instar larval ring gland from a 3R:24.4M fly immunostained for lacZ and Wg. The large lateral cells of the prothoracic gland (PG) display strong nuclear LacZ staining. Wg protein can be seen at the cortical surface of these cells. (C, C′) Confocal images of ring glands from *phm*Gal4 (C) or *Phm*Gal4>TCF^DN^ (C′) ring glands containing the 3R:24.4M transgene immunostained for lacZ and DAPI. TCF^DN^ expression results in a dramatic reduction in lacZ expression in The PG. (D) Normalized pixel intensity of the LacZ reporter was calculated for 120 nuclei representing each condition, and the data were summarized using a Tukey box plot, with outliers represented by circles.

*fd96Ca* and *b* transcripts were previously reported to be expressed in 14 pairs of ventral stripes after germband extension [72]. To determine whether this expression was dependent on Wg signaling, we examined expression in embryos where Wg signaling was inhibited. Using probes designed to unique regions of the *fd96Ca* and *b* transcripts, we determined that *fd96Cb* was expressed in ventral stripes, reminiscent of the W-CRM expression pattern, and that this expression was greatly reduced or lost in *da*-Gal4>*arm*RNAi and *da*-Gal4>TCF^DN^ embryos (Figure S9). Our results strongly suggest we have identified a W-CRM that is required for Wg-dependent activation of *fd96Cb* in embryos.

The second putative W-CRM is located at chromosomal position 3R:24.4M, in the 3′ UTR of the *forkhead* (*fkh*) gene. In 3^rd^ instar larvae, a lacZ reporter containing this element was strongly expressed in the PG, a part of the ring gland (Figure 9B, middle panel). Although the PG had not been previously linked to Wg signaling, Wg protein was clearly detectable in this tissue by immunostaining using two independent antibodies (Figure 9B, S10). To confirm that the 3^rd^ instar expression pattern was dependent on Wg signaling, the PG-specific *phantom* (*phm*)-Gal4 driver [73] was used to drive TCF^DN^. A tub-Gal80^ts^ transgene was included [74], so that expression of TCF^DN^ was limited to 24 hr prior to dissection and staining. This treatment resulted in a dramatic reduction of lacZ expression compared to controls in late 3^rd^ larval instars (Figure 9C′). The reduction is quantified in Figure 9D. These results indicate that the 3′ UTR of *fkh* contains a PG-specific W-CRM.

*fkh* has been previously shown to be downstream of Wg signaling in the salivary placode [75] and has been shown to be required for the maintenance of Wg expression in the developing hindgut [76]. Combined with the promixity of the 3R:24.4M W-CRM to the *fkh* promoter, this suggested that *fkh* might be a Wg target in the PG. However, using an anti-Fkh antisera [77], we found no detectable expression of Fkh in the ring gland, suggesting that the 3R:24.4M W-CRM may act at a distance to regulate expression of another gene.

While the identity of the gene(s) regulated by the 3R:24.4M W-CRM is not clear, our finding that the reporter is dramatically inhibited by expression of TCF^DN^ suggests that Wg signaling may play a role in ring gland biology. Consistent with this, when TCF^DN^ is expressed via the *phm*Gal4 from embryogenesis on, developmental arrest occurred during the first larval instar with 100% penetrance. These results argue that Wg signaling has a previously unappreciated role in the development of the ring gland.

## Discussion

### The Rules of TCF/Pan Binding to HMG-Helper Site Pairs

Previous work has shown that TCFs containing C-clamp domains recognize two distinct DNA sequence motifs, HMG sites (via the HMG domain) and Helper sites (via the C-clamp) [44]–[46], [48], [78]. The close proximity of these motifs suggested that they act as HMG-Helper site pairs, which we confirmed through site-directed mutagenesis (Figure 1C). Since HMG and Helper sites are often clustered in W-CRMs (Figure 1A), it was not readily apparent what orientation and spacing constraints exist for these sites to form a functional bipartite TCF binding site. In this report, we employed a variety of approaches to determine which HMG-Helper site configurations enhanced TCF/Pan binding in vitro and in vivo, and which ones allowed transcriptional activation by Wnt/β-catenin signaling.

Our analysis revealed that HMG-Helper pairs in the FF0 and AK6 arrangement are preferred in a number of situations. These configurations were bound by TCF/Pan with the highest affinity in vitro (Figure 2) and were highly enriched in chromatin bound by TCF/Pan in embryos (Figure 6). In cell culture, synthetic reporters with FF0 and AK6 pairs were the most highly activated by Wnt signaling (Figure 3A). Similar results were also obtained in transgenic reporter assays in several imaginal discs (Figure 4). These results demonstrated a strong correlation between DNA binding affinity of HMG-Helper pairs for TCF/Pan and their ability to mediate Wnt-dependent activation of transcription in several contexts.

While the aforementioned data support the view that some HMG-Helper site configurations are better than others, additional analyses paint a more complex picture. In the context of endogenous W-CRMs, FF1 and AK6 were also the most active in promoting transcriptional activation, but AK1 was just as good in some contexts (Figure 3B, 3C). This dovetailed well with the computational analysis of TCF/Pan ChIP-Seq data, where AK0-6 showed the highest enrichment for this orientation (Figure 6). However, AK0 showed only moderate affinity in vitro (Figure 2), similar to other configurations (KK0, FF6, RW6) which had reduced or no functional activity in synthetic reporters in cultured cells (Figure 3A) and imaginal discs (Figure 4, 5H). The correlation between DNA binding affinity and transcriptional activation was poorest in the larval epidermis and CA, e.g., RW0 and KK6 drive robust activity in these tissues despite being weakly bound in vitro, while higher affinity motifs drive much weaker expression. A disconnect between in vitro binding affinity and transcriptional activation in cells has also been observed for glucocorticoid receptor [79]. This work and our data demonstrate that some caution is needed when inferring functional significance from in vitro binding studies.

Another general lesson from our work is that the presence of a Helper site near a HMG site, no matter the orientation, increased TCF/Pan binding affinity and its ability to mediate Wnt activation of transcription. This is evident in the EMSA data, where all eight HMG-Helper pairs were bound with greater affinity than HMG sites alone (Figure 2), and in TCF/Pan bound chromatin, where enrichment of HMG-Helper pairs was observed over a surprisingly wide array of orientation/spacings (Figure 6). This flexibility was also observed functionally in the synthetic reporters, where HMG site alone constructs had no detectable expression but all eight HMG-Helper site configurations tested had detectable reporter activity in some tissues (Figure 5H).

**Figure 10:**
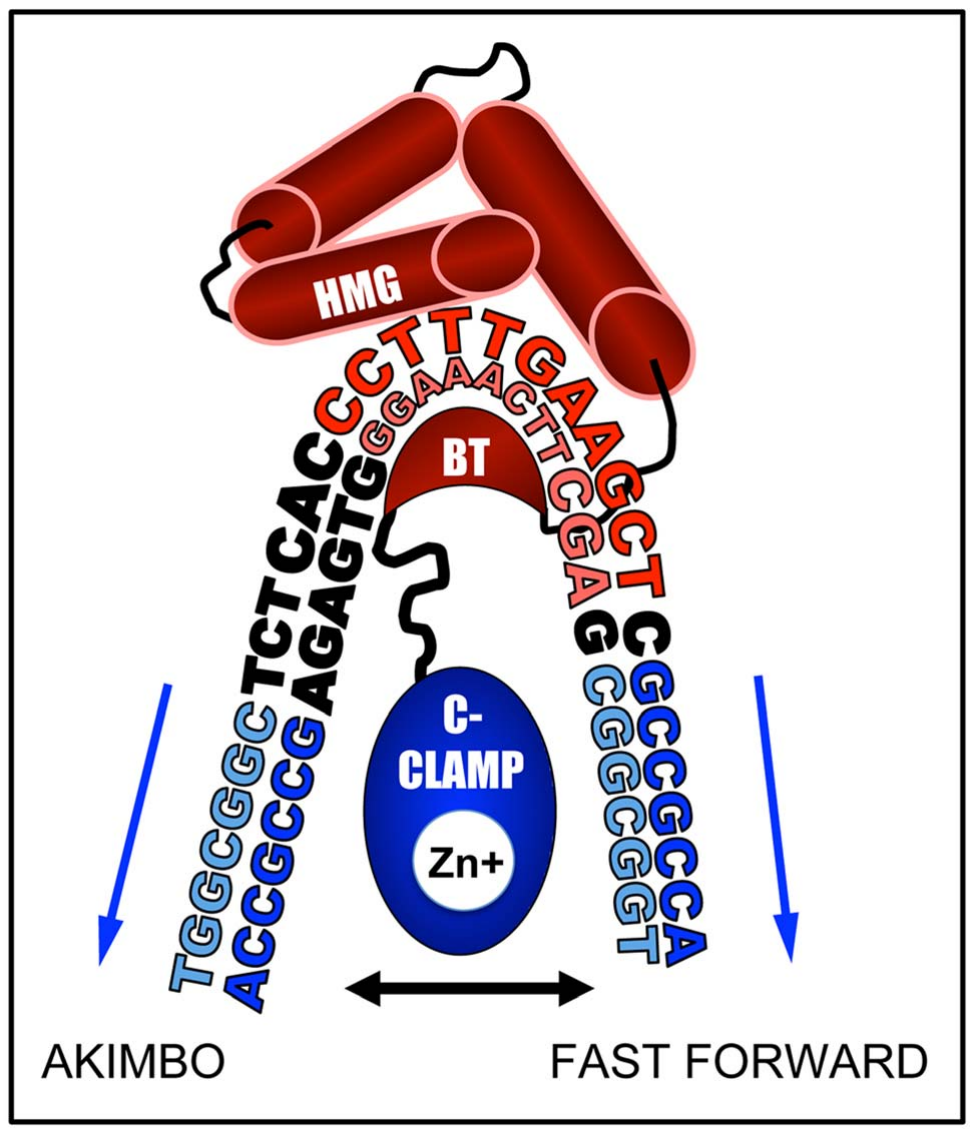
DNA bending by the HMG domain could explain preferential binding for AK6 and FF0 configurations. A cartoon based on the NMR-deduced structure of the HMG domain and basic tail (BT) of murine LEF-1 [80] bending the HMG site. The HMG domain is composed of three alpha-helices (red barrels), the first of which binds the minor groove of the HMG site (shown in red), while the BT (red crescent) wraps around to make contact with the major groove. This binding induces a sharp bend in the DNA, most pronounced between positions T3 and T4 (CC**TT**TGATCTT). In TCF/Pan, which shares 92% identity with the HMG domain of LEF1, the BT is followed by a 10 residue linker, and then by the C-Clamp (blue oval), recently shown to chelate a zinc ion [48]. The C-clamp can bind to Helper sites (blue sequences) either upstream (AK) or downstream (FF) of the HMG site. We postulate that the linker places a constraint on the optimal spacing for the FF and AK orientation of 0 and 6 bp, respectively.

How can the HMG and C-clamp domains, which are separated by only ten amino acid residues, bind to HMG-Helper pairs with such diversity? We think it likely that DNA bending by TCF/Pan is a major contributor to this flexibility of DNA recognition. Murine LEF1 has been shown to bend DNA more than 110° [80] and TCF/Pan possesses a similar ability [33]. The C-Clamp is located 10 amino acids C-terminal to the basic tail (BT) in TCF/Pan [1], which may place the C-clamp in the interior of the DNA bend, allowing it to “swing”, and interact with Helper sites located either “upstream” of the HMG binding site (AK) or “downstream” (FF) (Figure 10). The bend is centered between the third and fourth position in the eleven bp HMG site, placing Helpers in the FF orientation further away from the C-terminus of the basic tail (BT) of TCF/Pan (Figure 10). This could explain why FF0 was bound preferentially over FFs with larger spacing between the HMG and Helper sites. Conversely, AK6 may be bound with highest affinity (at least in vitro) compared to AK0 due to less steric hindrance from the amino acids connecting the BT and the C-clamp (Figure 10).

In addition to DNA bending, the semi-palindromic nature of the Helper site likely explains why KK and RW configurations also enhance TCF/Pan binding (Figure 2 & 6) and have transcriptional activity (Figure 3A, 4 & 5). For example, the KK0 sequence (HMG site-TGGCGGCG ) can also be viewed as a degenerate FF1, with a C to G substitution at positions 2 and 5 of the Helper site (Figure 1D). The same is true for the RW configuration (e.g., RW0 could be a degenerate AK1). Viewed in this way, the IC_50_ data becomes more coherent, with the FF and KK configurations ranked FF0>KK0>FF6>KK6 in terms of affinity for TCF/Pan and the AK and RW ones ranked AK6>RW6>AK0>RW0 (Figure 2B). Defining KK and RW as degenerate FF and AK orientations, respectively, can explain why these motifs mirror the spacing constraints of their reverse configuration partners, and why they are bound with weaker affinity and typically display less transcriptional activation activity.

### Biological Relevance of HMG-Helper Site Configurations for Wnt Gene Regulation

In the wing imaginal disc, Wg has been proposed to act as a morphogen, forming a concentration gradient emanating from the dorsal/ventral boundary and regulating target gene expression in a concentration-dependent manner [81]–[84]. How W-CRMs differently respond to this Wg morphogen gradient has not been previously investigated. To address this important question, we utilized the *nkd*UPE2 reporter, which is activated in areas of high Wg ligand concentration in the wing disc [57]. Replacing a low affinity RW4 motif in this W-CRM with a high affinity AK6 motif elevated the level of reporter gene expression, and broadened the expression domain (Figure 7). These results argue that increasing the affinity of TCF/Pan for the W-CRM increases the sensitivity of the W-CRM to respond to the Wg morphogen.

Our data are reminiscent of classic studies of CRMs that are controlled by gradients of TFs in the syncytial blastoderm stage of *Drosophila* embryogenesis. The affinity of the binding sites for the *c-rel* homolog Dorsal has been shown to set threshold responsiveness in dorsal/ventral patterning, with higher affinity sites being more sensitive to the Dorsal gradient [85]. In contrast, higher affinity sites have been shown to restrict the domain of expression of CRM reporters for the transcription factor Cubitus Interruptus (Ci), an effector of Hedgehog signaling [86], [87], possibly due to homo-cooperative interactions with the repressive form of Ci [87], [88]. Although Ci and TCF/Pan both act as transcriptional switches, our study indicates that the relationship between binding site affinity and interpretation of the signaling gradient are diametrically opposed for these two factors.

Another interesting feature of our work is the tissue-specific responses of our synthetic HMG-Helper site reporters in transgenic fly tissues. In imaginal discs, the strength of expression of these reporters was largely correlated with binding affinity (Figure 4, 5H). However, low affinity RW and KK motifs, which had little or no activity in imaginal tissues, drove robust expression in the larval epidermis and the CA cells of the ring gland (Figure 5C,F). Given that these simple reporters presumably only contain TCF/Pan sites plus a minimal promoter, the data suggest that TCF/Pan is allosterically regulated by DNA in a tissue-specific manner. Allosteric regulation of TFs by their cognate binding sites is known to occur [52], [55], [79], [89], [90], and has been proposed previously for TCF/Pan [32], [33]. In these cases, the type of DNA binding site is thought to control whether the TF activates or represses transcription. Our data suggest an additional aspect of allosteric regulation of TCF, i.e., TCF/Pan bound to different HMG-Helper pairs may allow interactions with distinct co-regulators, which enable it to activate transcription in a tissue-specific manner.

The aforementioned data demonstrates that different HMG-Helper pairs can profoundly influence the strength and/or tissue-responsiveness of promoters to Wnt signaling. While this was only examined in detail for a handful of reporters, our computational analysis supports the view that HMG-Helper pairs of all four orientations and various spacings contribute to TCF/Pan binding to chromatin (Figure 6, S4, S5). Therefore, we speculate that there are many other such examples in the genome, and that the flexibility of TCF/Pan to HMG-Helper pairs provides a versatile evolutionary mechanism for CRMs to modulate their response to Wnt signaling.

### C-Clamp Containing TCFs in Other Systems

The genome sequences of many metazoans indicates that almost all invertebrates have a single TCF containing a C-Clamp, while vertebrates have four or more TCFs, with E-tail isoforms of the TCF1 and TCF4 genes containing a C-clamp [1], [21]. While the HMG and C-clamp domains are highly conserved in most metazoans, POP-1, the *C. elegans* TCF, is somewhat divergent [1]. Perhaps more importantly, the linker sequence between the HMG and C-clamp domains is variable, ranging from 5–40 aa, e.g., it is 23 aa in human TCF1E, compared with 10 aa in TCF/Pangolin and 9 aa in POP-1 [1]. These differences could influence the rules for preferred HMG-Helper site configurations in different organisms.

Despite these concerns, the available data suggests that other metazoans have a similar bias for HMG-Helper pair configurations as we have found in *Drosophila*. We have recently characterized four W-CRMs in *C. elegans*, identifying a functionally important HMG-Helper pair in each one. Three of these were FF orientations of 0, 1 & 2 spaces, while the fourth is an AK7 [78]. Furthermore, in a search for new *C. elegans* W-CRMs, 3 putative modules containing HMG and Helper clusters were chosen based on sequence conservation and individual site quality, however, only the module containing an optimal motif (AK7) was bound by POP-1 in an in vitro binding assay [78]. These results suggest that the rules for POP-1 DNA binding share important similarities with TCF/Pan.

In humans, an in vitro protocol for enriching preferred sequences flanking an HMG site for TCF1E reveals Helper-like motifs (RCCG) that are bound by the C-clamp [45], [46]. This consensus is shorter than the Helper motif we identified in flies (GCCGCCR) [44]. However, the functional Helper sites identified in several W-CRMs that are activated by TCF1E in a colon cancer cell line share the consensus GCCGCY [46], consistent with human Helper sites containing at least six nucleotides. In regard to HMG-Helper site spacing/orientation, the in vitro studies found preferred binding with either AK2-9 or FF0-11 configurations [46]. Systematic mutagenesis of Helper sites in the *Sp5* W-CRM revealed three functional HMG-Helper pairs with configurations of AK7, RW1 and FF1, and other W-CRMs that were Helper site-dependent had predominately FF and AK configurations [46]. While analysis of additional W-CRMs in flies, worms, humans and other systems is required, the general rules for TCF-DNA recognition outlined in this report clearly provide a strong foundation for further studies.

### *In Silico* Identification of Novel W-CRMs

The high level of degeneracy in TCF binding sites [91] makes in silico detection of W-CRMs difficult. The use of evolutionary conversation can facilitate such searches, e.g., the EEL algorithm [92]. We previously demonstrated that searching for clusters of HMG and Helper sites in the fly genome could identify W-CRMs that are directly activated by Wnt signaling in cell culture [44]. In this report, we incorporated the knowledge gained from analyzing the functional architecture of HMG-Helper site pairs to refine our computational searching. Our basic strategy employed searching the genome for high quality “optimal conformation” HMG-Helper pairs, followed by secondary searches for nearby lower quality pairs, which resulted in the identification of several novel W-CRMs.

We utilized the aforementioned strategy to screen chromosome 3R for high quality FF1 pairs. This analysis revealed stretches containing multiple HMG-Helper pairs near the *fkh* and *fd96C* loci, which also possessed W-CRM activity in embryos and the ring gland (Figure 8, 9). Our results indicate that searches biased for those HMG-Helper site configurations that are bound by TCF/Pan with highest affinity in vitro can successfully identify novel W-CRMs.

Given our functional data that other “non-optimal” HMG-Helper pairs can also recruit TCF/Pan and promote Wnt-dependent transcription, often in tissue-specific ways (Figure 4, 5), additional searches for these configurations should be a useful approach for W-CRM identification. For example, the *mab-5* gene in *C. elegans* is a known target of Wnt signaling [93], but a W-CRM in its regulatory DNA had not been identified [94]. Using our search protocol, we identified a FF7 pair 9.4 kB upstream of the *mab-5* ATG, which was demonstrated by others to have W-CRM activity in mab-5 expressing cells [94]. Expression of this reporter was significantly reduced by mutation of the HMG site identified by our search [94]. These HMG and Helper sites are fairly divergent (i.e., TCTTTTGCCTC & GCCATAA) which highlights another application of the results in our report: functional TCF sites that diverge from the consensus can still be identified if HMG-Helper site pairing is considered, as long as the amount of DNA to be searched is not too extensive (e.g., <12 kb).

Computational searching for HMG-Helper pairs offers a complimentary approach to genome-wide surveying of TCF/Pan binding using ChIP-seq. While the region containing the *fd96c* W-CRM was identified as a TCF/Pan-bound region in fly embryos [28], the 3R:24.4M W-CRM was not, highlighting the limitation of using one source of material for ChIP-seq analysis. On the other hand, while computational analysis of HMG-Helper pairs may help to prioritize which TCF/Pan ChIP-seq peaks might be functionally relevant, it is also likely that TCF/Pan is recruited to many W-CRMs by protein-protein interactions, given that HMG-Helper pair enrichment is markedly reduced in TCF/Pan-bound regions that are also occupied by other TFs (Figure 6B, S5).

Despite our success with *in silico* identification of W-CRMs, our results indicate that connecting these W-CRMs with endogenous targets may not be straightforward. In the case of the W-CRM in the *fd96C* locus, part of its pattern is very similar to that of endogenous *fd96Cb* expression. However, the W-CRM reporter is also expressed in other parts of the embryo (Figure 8, S9), possibly because the *fd96C* locus contains other inhibitory CRMs that refine gene expression, as has been found for other genes [95]. For the W-CRM found in the 3′ UTR of the *fkh* gene which is highly active in the PG (Figure 9), we found no evidence for endogenous Fkh expression in this tissue. Given that CRMs can act at great distances and pass over nearby promoters in *Drosophila* and vertebrates [96]–[99], it is possible that this W-CRM regulates other gene(s) on chromosome 3R.

Another benefit of *in silico* based discovery of W-CRMs is highlighted by our identification of the 3R:24.4M W-CRM, which is expressed in the PG cells of the ring gland (Figure 9C, 9C′, 9D). This endocrine organ is a master regulator of *Drosophila* molting behavior [100], [101], but had not been previously linked to Wnt signaling. Wg protein was detected on PG cells (Figure 9B, S10), and transient inhibition of Wg signaling in the PG results in reduced expression of the 3R:24.4M W-CRM reporter in third larval instar (Figure 9). Wg activity in this tissue is biologically important, because constitutive disruption of the Wg pathway results in developmental arrest during first larval instar, presumably due to the inability to molt. Interestingly, some synthetic HMG-Helper pairs (e.g., KK6) are highly active in the CA region of the ring gland and require Wg signaling for activity (Figure 5). Why the synthetic elements and the endogenous 3R:24.4M W-CRM are active in different cells of the ring gland is not clear. We are currently exploring the role of Wg signaling in ring gland biology and think it likely that computational searches for W-CRMs will uncover additional roles for the Wg pathway in other tissues.

## Materials and Methods

### Plasmids

Synthetic HMG-Helper pairs were synthesized by Integrate DNA Technologies (IDT; Coralville, IA) and cloned into a modified pGL3-Basic vector (Promega) containing an *hsp70* minimal promoter [32] for cell culture assays, or the pLacZattB vector [59] for transgenic fly generation, using BglII and XhoI restriction sites. The *nkd*IntE and *nkd*UPE2 reporter gene vectors were described previously [44], [57], and mutagenesis was carried out using the Stratagene QuickChange kit (Agilent). For the *fd96C*Mid and *fkh*3′UTR W-CRMs, the fragments were amplified using Roche High Fidelity enzyme, using w^118^ genomic DNA as the template, and cloned into TOPO TA (Invitrogen) as an intermediate before being moved into the pLacZattB vector, using the Acc65I and NotI sites. pAcArm* and p*arm*LacZ have been described previously [32], [44], [57]. The protein expression vector for EMSA was generated by cloning the region encoding the HMG domain and the C-clamp into the XmaI and SacI restriction sites of the pET52b(+) vector (Merck Millipore).

### Cell Culture

*Drosophila* Kc167 cells were cultured in Schneider's *Drosophila* Medium (Gibco) supplemented with 10% Fetal Bovine Serum (Gemini Bioscience). 250 ul of cells were seeded in 48 well plates, at a density of 1million cells/ml, and transient transfections were performed using Fugene transfection agent (Roche). Each well received 20 ng luciferase reporter vector and 2 ng pArmLacZ. Wnt signaling was activated by transfection with 10 ng pAcArm*, (a constitutively active Arm protein), and pAC5.1 EV was used as filler DNA to 100 ng total for each well. Cells were lysed and treated three days later using the Tropix Luc-screen kit (Applied Biosciences) and Luciferase and LacZ activity assayed using the Promega Glomax system. pArmLacZ was used to normalize for transfection efficiency.

### EMSA

A His-tagged fragment of TCF/Pan containing both the HMG and C-Clamp domains was purified from *E.coli* strain BL21 following IPTG induction for 4 hours @ 37° using column purification on Nickel beads (Invitrogen) with Immidazole elution. LB growth media supplemented with 10 uM ZnCl. dsDNA probes were purchased from IDT and labeled probe was tagged with a 5′ 700 IR moiety on both strands. Competition assays were performed using the LI-COR Odyssey Infrared platform, and infrared intensity of the IR dye-labeled probe/protein complexes were calculated using Image Studio 2.0. The IC_50_ values were calculated using Prism 6 for Mac OS X (Graphpad Software, La Jolla California), as were the saturation binding curves. Three independent experiments were used to perform a least-squares non-linear fit. Binding reactions were performed as described in [44], briefly, with 50 ug/ml poly(dIdC). 0.05% NP40, 50 mM MgCl2 and 3.5% glycerol in binding buffer (10 mM Tris-HCl, pH 7.5, 50 mM KCl, 1 mM DTT). Each reaction, containing 6 pmol recombinant protein and 0–2.4 pmol competitor dsDNA (dose indicated in figure 4A) was incubated for 5 min on ice, 25 minutes at RT before 20 fmol IR-dye labeled probe was added and reactions were incubated for an additional 30 minutes. A complete list of the probes used can be found in Table S1.

### *Drosophila* Genetics

Synthetic and endogenous W-CRMs were cloned into the pLacZattB vector [59] and injected by Rainbow Transgenics (Camarillo, CA) using a φ-C31 site directed integration strategy. All constructs were injected into line 24749, integration site 86Fb. 1–3 individual lines were analyzed for each construct, and as expected, no variation in expression level or pattern was seen between lines. Candidate W-CRM constructs were recombined with UAS lines expressing a dominant negative TCF/Pan [58] or an *arm*RNAi hairpin [102] and crossed to the appropriate GAL4 driver line using standard techniques. *da*Gal4 [68] was used to drive expression in the embryonic epidermis, while the ring gland-specific driver *phm*GAL4 (created by M. B. O'Connor) was obtained from Michael Stern. The CA-specific driver *Aug21*Gal4 [103] was obtained from the Bloomington Stock center.

### Imaging of *Drosophila* Tissues

Cuticle preparations were performed as previously described [71]. To detect β-galactosidase activity, third-instar larval discs were fixed in 1% gluteraldehyde (in PBS), and incubated in staining solution (10 mM NaPO_4_, 150 mM NaCl, 1 mM MgCl_2_, 6 mM K_4_[Fe^II^(CN)_6_], 6 mM K_3_[Fe^III^(CN)_6_], and 0.3% Triton X-100, plus 2 mg/ml X-gal) for 25 min at room temperature. After the reaction was stopped, discs were mounted in 70% glycerol. Images were taken on a Nikon Eclipse E600 upright microscope with Spot basic software and processed using Gimp v2.8 or Adobe Photoshop CS5.1. Immunostaining was performed as described in [104], using rabbit anti-LacZ (MP biomedicals) and mouse anti-Wg concentrate (Developmental Studies Hybridoma Bank, University of Iowa). Embryos were collected for 24 hours before processing, and both antibodies used at a dilution factor of 1∶1200. For the PG, larvae were collected at the third instar larval phase, and a 1∶600 dilution of each antibody was used. For all samples, CY3 (Jackson Immunochemicals) and Alexa 488 (Molecular Probes) conjugated secondary antibodies were used at a 1∶300 dilution. Affinity purified rabbit anti-Wingless antisera was used at a 1∶20 dilution. Images were taken on a Leica DM6000B confocal microscope and processed using Gimp v2.8 or Adobe Photoshop CS5.1. 1–3 individual lines were analyzed for each construct, and representative images are shown. Normalized pixel intensity was calculated using Leica LAS software to measure pixel intensity in bounded nuclei. Mean LacZ fluorescent intensity for each nucleus was normalized to mean DAPI fluorescent intensity and Tukey box plots were generated using open source software (http://boxplot.tyerslab.com/). For in situ hybridizations, digoxigenin-labeled RNAprobes were designed to unique regions in *fd96Ca* and *b* (see Table S1 for sequences) and hybridizations were carried out following the protocol outlined in [105].

### Bioinformatic Analysis of ChIP Seq Data

Training sequences for PWMs were taken from previously defined functional sites in W-CRMs depicted in Figure 1A. PWM scores were calculated using the formula: weight_i,j_ = ln{[(n_i,j_+p_i_)/(N+1)]/p_i_}∼ln(f_i,j_/p_i_). The high confidence TCF/Pan bound regions [28] were searched for bipartite motifs and binned according to orientation and spacing using the dm3 genomic assembly in Matlab. To generate a random set of DNA sequences to analyze, an aggregate list of all sequences found in the 5′, intergenic, intronic, and 3′ data sets was created. Each sequence from the set was assigned an index 1 through N, where N was the index of the last sequence in the aggregate set. A random ordering of all indices was then created and used to iterate over the data set, thus guaranteeing the same sequence could not be selected more than once. For each iteration, if a sequence contained a minimum size of 50 base pairs it was analyzed using the same processes as was used on the target data set. When the number of random sequence base pairs equaled or exceeded the number of base pairs in the target data set, the random data analysis was concluded. For each run of the random sequence analysis, the random number generator was seeded such that successive runs did not analyze the same fragments.

## Supporting Information

Figure S1 Embryonic activity of synthetic HMG-Helper pair W-CRM reporters. Brightfield images of stage 10/11 (top of each panel) and stage 13 (bottom of each panel) embryos containing the indicated lacZ reporter constructs stained for lacZ activity. In all HMG-Helper pairs tested, little expression was observed.
(TIF)

Figure S2 Wg expression in imaginal discs. Confocal images of wing (A) and eye/antennal and leg (B) imaginal discs immunostained with a rabbit affinity purified anti-Wg antibody.
(TIF)

Figure S3 Position weight matrices for HMG and helper sites. Training sequences for matrixes shown to the left. Weighted scores (in bold on right) were calculated using the formula weight_i,j_ = ln{[(n_i,j_+p_i_)/(N+1)]/p_i_}∼ln(f_i,j_/p_i_). Sequence logos shown below the position weight matrixes were designed using Weblogo (http://weblogo.berkeley.edu/).
(TIF)

Figure S4 Enrichment of HMG-Helper pairs in TCF/Pan-bound DNA using a low stringency calling criteria. A calling criteria of 3.5 for HMG site and 5.0 for Helper sites (based on the position weight matrixes shown in Figure S1) was used to identify HMG-Helper pairs in TCF/Pan bound and random DNA (see Figure 7 and text for further explanation). HMG-Helper pairs are ∼2.4 times more likely to occur in TCF/Pan bound regions than in random DNA (2139 hits vs 893.1).
(TIF)

Figure S5 HMG-Helper pairs are less enriched in TCF/Pan bound regions shared by other cardiogenic TFs. TCF/Pan bound peaks were divided into groups based on whether the center of the peak was located within 150 bp of the peak for another TF. HMG-Helper pair enrichment is much greater in “unique peaks” than in shared peaks. The difference is especially evident in the FF0-2 and AK0-6 range. A subset of this data is shown in Figure 7B.
(TIF)

Figure S6 The optimized *nkd*UPE2 reporter is Wg signaling dependent. (A,B) Bright field images of wing imaginal discs from late 3^rd^ larval instar from animals containing the optimized *nkd*UPE2 reporter, *Dpp*-Gal4, TubGal80ts without (A) or with (B) UAS-TCF^DN^. Animals were shifted from 18°C to 29°C for **48 hr** prior to dissection. The reporter gene is severely repressed at the anterior posterior boundary, where the Dpp-Gal4 is active.
(TIF)

Figure S7 *In silico* search for FF1 HMG-Helper site pairs. (A) Training sequences and PWM used in a search of Chromosome arm 3R. (B) List of the top 23 hits, location and genomic environment.
(TIF)

Figure S8 Da-Gal4>UAS-ArmRNAi embryos secrete cuticle with hallmarks of reduced Wg signaling. Darkfield micrographs of end stage embryo cuticles from animals containing a P[*da*-Gal4] transgene and control (A) or P[UAS-*arm*RNAi] chromosome (B). The Arm depleted embryos have extra denticles indicative of a partial loss of Wg signaling [63], [71], but lack the gross abnormalities associated with a loss of cell adhesion [69], [70].
(TIF)

Figure S9 *fd96Cb* is activated by Wg signaling in the embryo. Bright field images of stage 13 embryos with *in situ* hybridization using dioxigenin-labeled probe complementary to the *fd96Cb* transcript. (A) Control embryos exhibited ventral stripes, similar to the *fd96C* W-CRM reporter. This expression was lost (B,C) or severely reduced (B′,C′) in embryos where *arm*RNAi (B,B′) or TCF^DN^ (C,C′) were ubiquitously expressed via the *da*-gal4 driver. The percentage of embryos displaying reduced or complete loss of signal is indicated in the bottom right corner of each panel (*arm*RNAi, n = 20; TCF^DN^ n = 52 embryos).
(TIF)

Figure S10 Wg expression in the ring gland using an affinity purified rabbit anti-Wg antisera. (B–B′) Confocal images of Wg immunostains (red) and DAPI (blue) demonstrating Wg expression in the ring gland (bottom two rows). Omitting the 1° antibody results in no signal (A).
(TIF)

Table S1 List of primers and probes used in cloning, site directed mutagenesis and EMSA binding assays.
(XLSX)
