# Supplementary figures and images for: Bipartite Recognition of DNA by TCF/Pangolin Is Remarkably Flexible and Contributes to Transcriptional Responsiveness and Tissue Specificity of Wingless Signaling
Source: PLoS Genet. 2014 Sep 4;10(9):e1004591. doi: 10.1371/journal.pgen.1004591 (PMC4154663; doi:10.1371/journal.pgen.1004591)

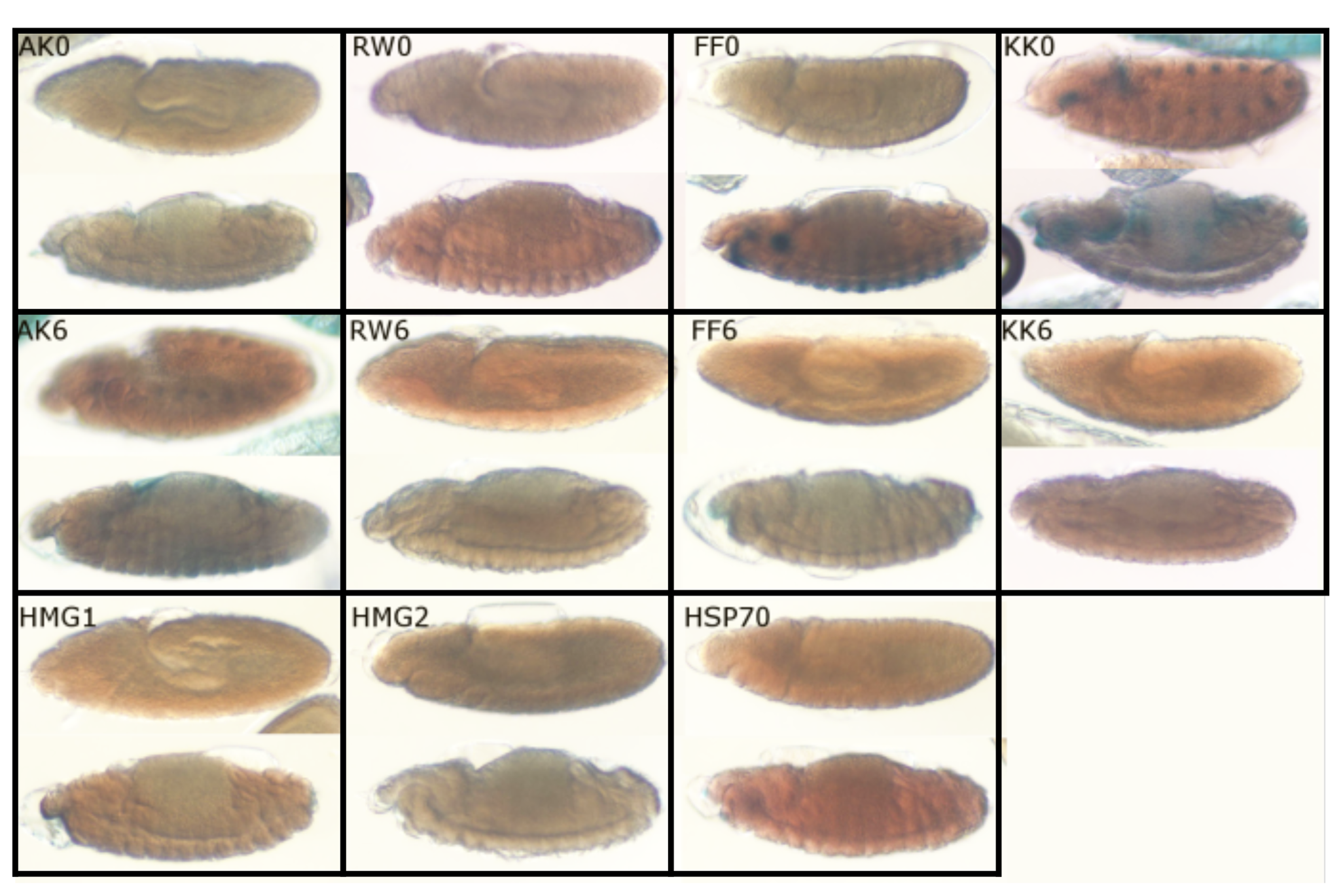

Supplement: Figure S1 — Embryonic activity of synthetic HMG-Helper pair W-CRM reporters. Brightfield images of stage 10/11 (top of each panel) and stage 13 (bottom of each panel) embryos containing the indicated lacZ reporter constructs stained for lacZ activity. In all HMG-Helper pairs tested, little expression was observed. (TIF) [file pgen.1004591.s001.tif]

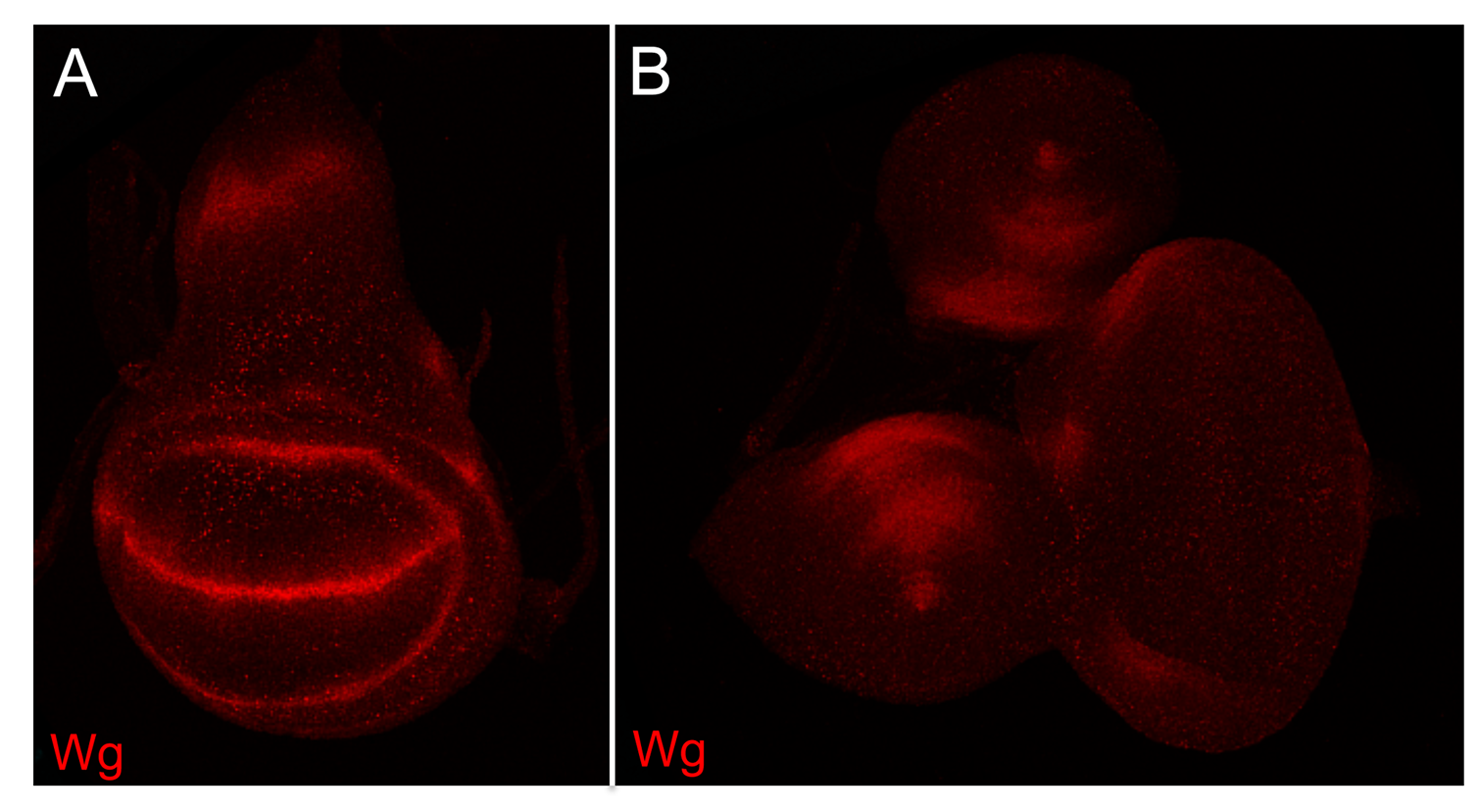

Supplement: Figure S2 — Wg expression in imaginal discs. Confocal images of wing (A) and eye/antennal and leg (B) imaginal discs immunostained with a rabbit affinity purified anti-Wg antibody. (TIF) [file pgen.1004591.s002.tif]

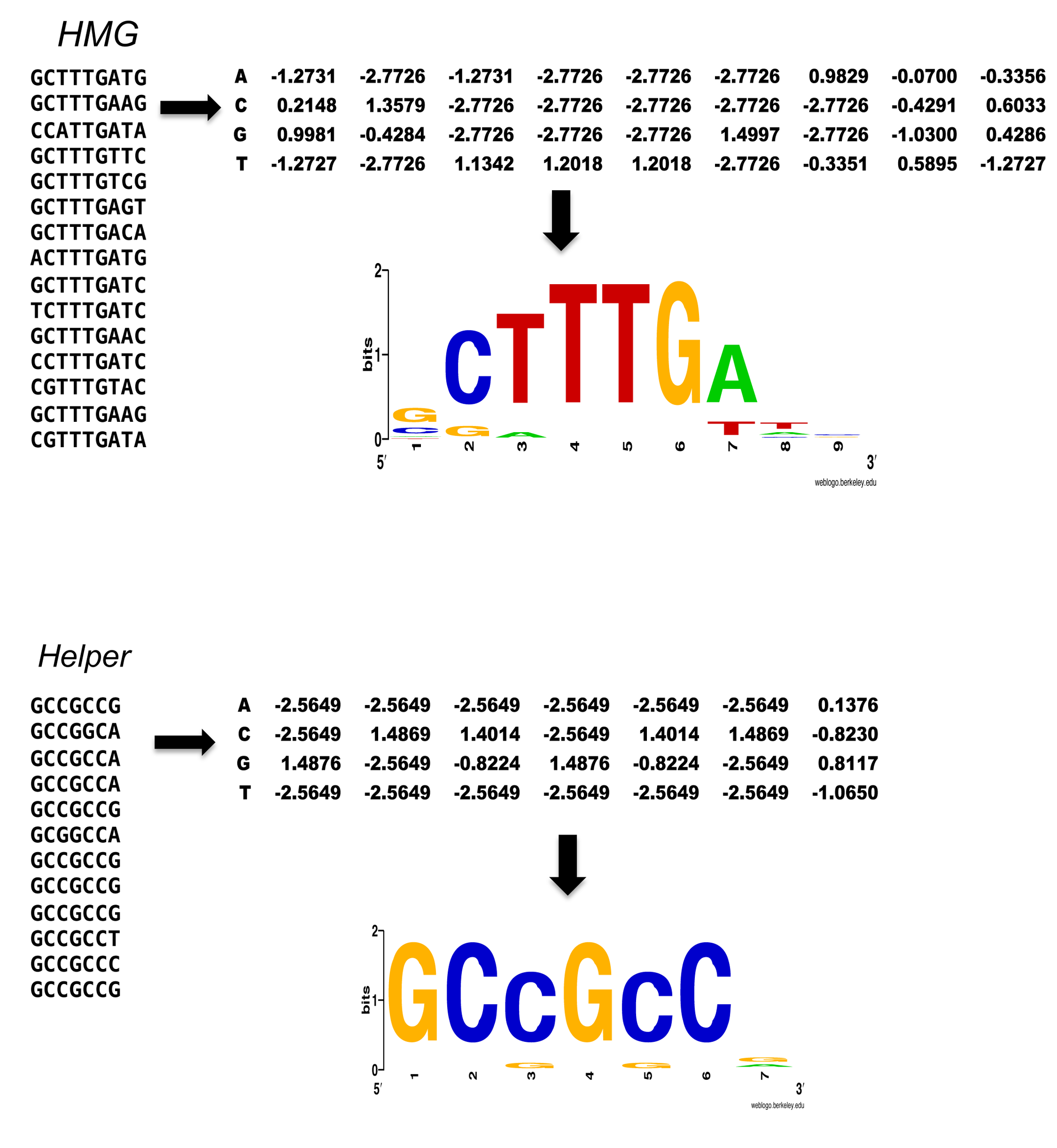

Supplement: Figure S3 — Position weight matrices for HMG and helper sites. Training sequences for matrixes shown to the left. Weighted scores (in bold on right) were calculated using the formula weighti,j = ln{[(ni,j+pi)/(N+1)]/pi}∼ln(fi,j/pi). Sequence logos shown below the position weight matrixes were designed using Weblogo (http://weblogo.berkeley.edu/). (TIF) [file pgen.1004591.s003.tif]

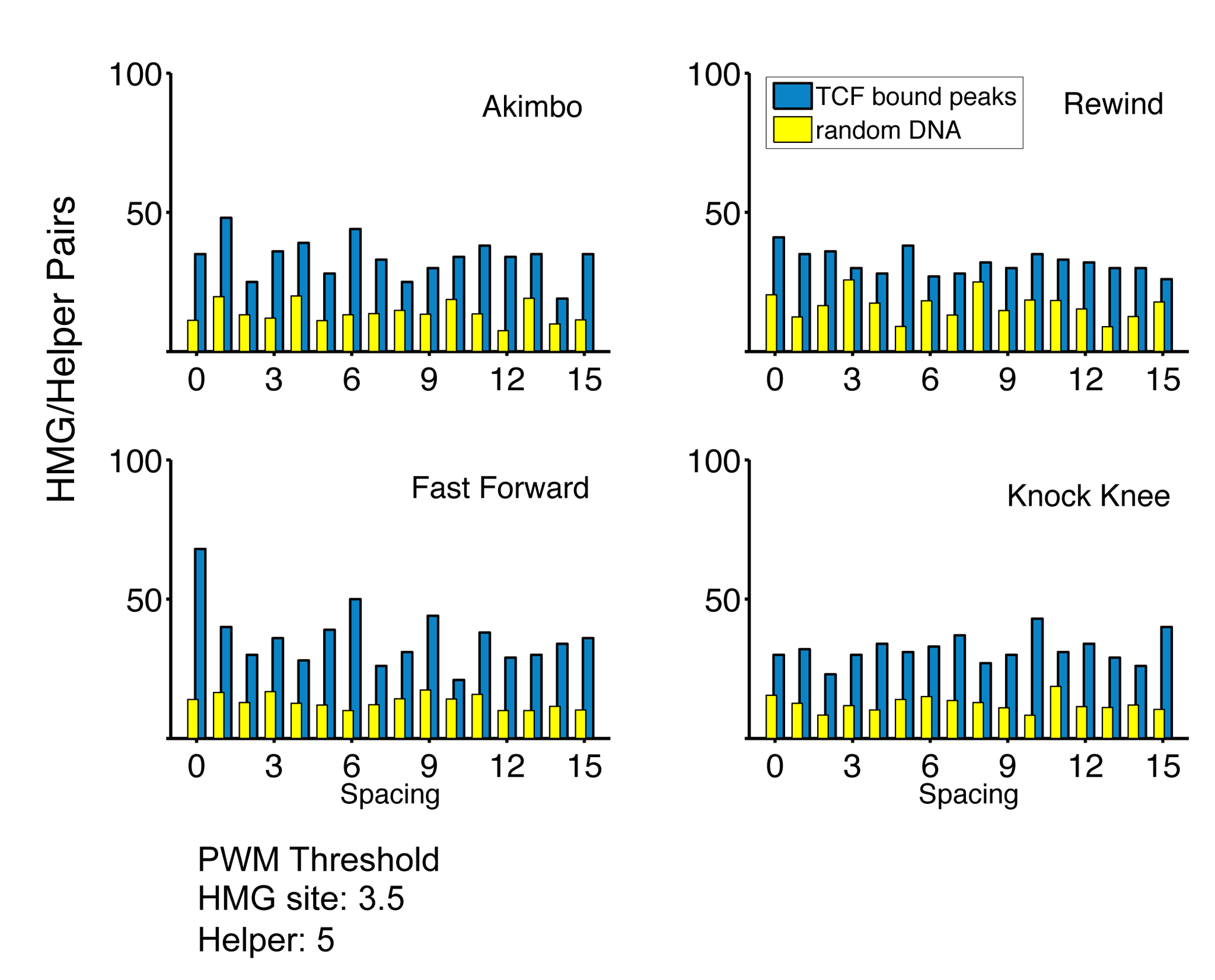

Supplement: Figure S4 — Enrichment of HMG-Helper pairs in TCF/Pan-bound DNA using a low stringency calling criteria. A calling criteria of 3.5 for HMG site and 5.0 for Helper sites (based on the position weight matrixes shown in Figure S1) was used to identify HMG-Helper pairs in TCF/Pan bound and random DNA (see Figure 7 and text for further explanation). HMG-Helper pairs are ∼2.4 times more likely to occur in TCF/Pan bound regions than in random DNA (2139 hits vs 893.1). (TIF) [file pgen.1004591.s004.tif]

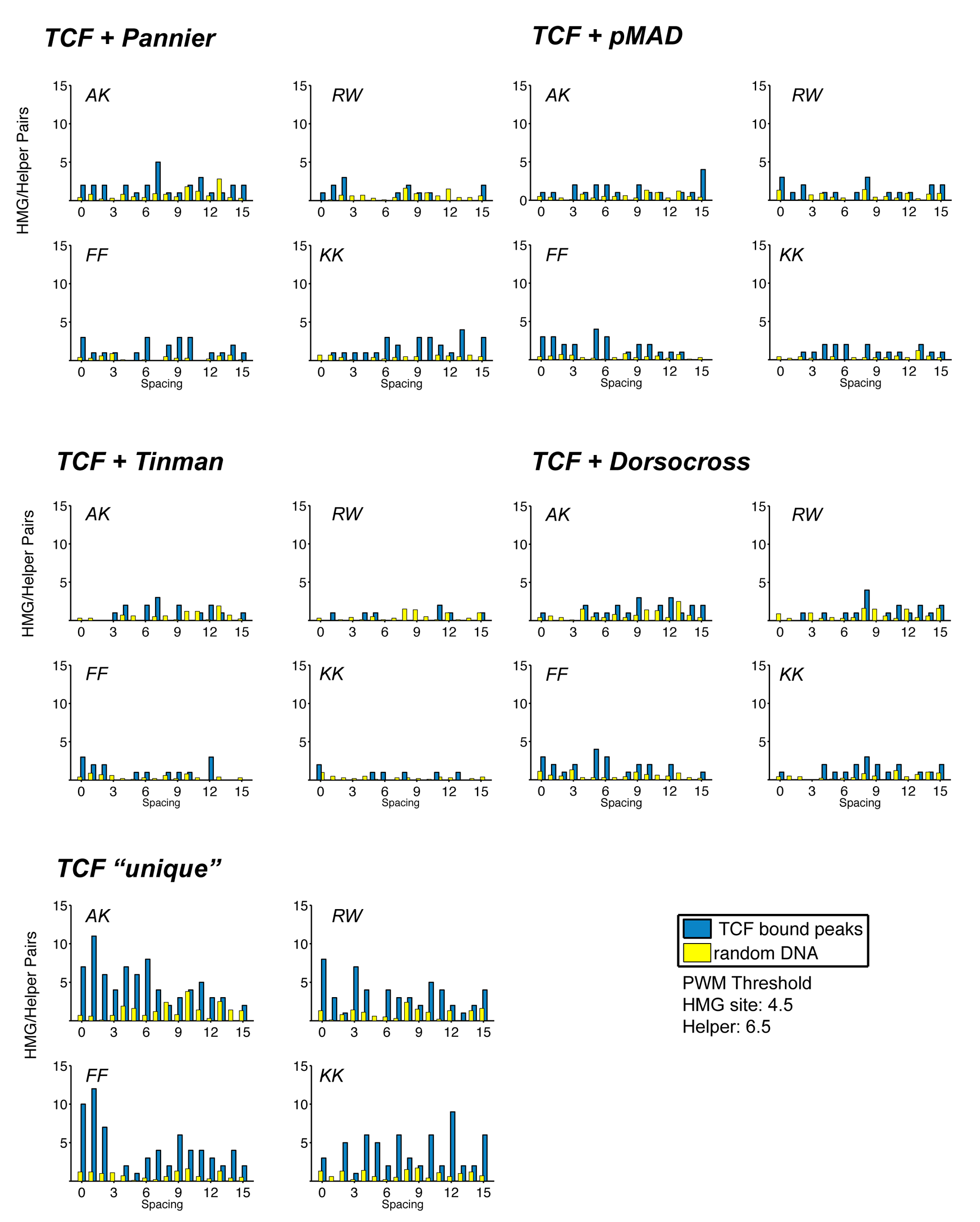

Supplement: Figure S5 — HMG-Helper pairs are less enriched in TCF/Pan bound regions shared by other cardiogenic TFs. TCF/Pan bound peaks were divided into groups based on whether the center of the peak was located within 150 bp of the peak for another TF. HMG-Helper pair enrichment is much greater in “unique peaks” than in shared peaks. The difference is especially evident in the FF0-2 and AK0-6 range. A subset of this data is shown in Figure 7B. (TIF) [file pgen.1004591.s005.tif]

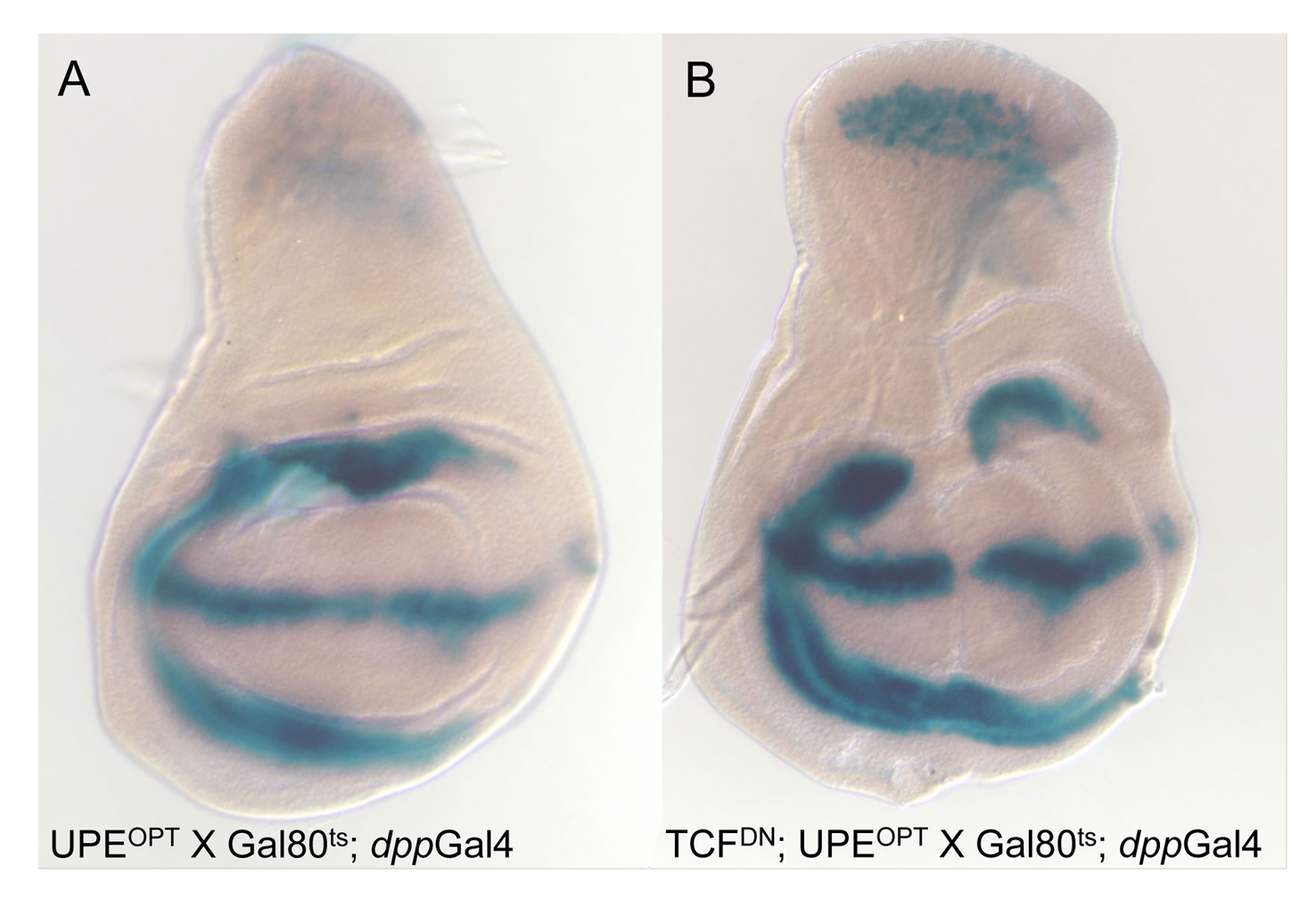

Supplement: Figure S6 — The optimized nkdUPE2 reporter is Wg signaling dependent. (A,B) Bright field images of wing imaginal discs from late 3rd larval instar from animals containing the optimized nkdUPE2 reporter, Dpp-Gal4, TubGal80ts without (A) or with (B) UAS-TCFDN. Animals were shifted from 18°C to 29°C for 48 hr prior to dissection. The reporter gene is severely repressed at the anterior posterior boundary, where the Dpp-Gal4 is active. (TIF) [file pgen.1004591.s006.tif]

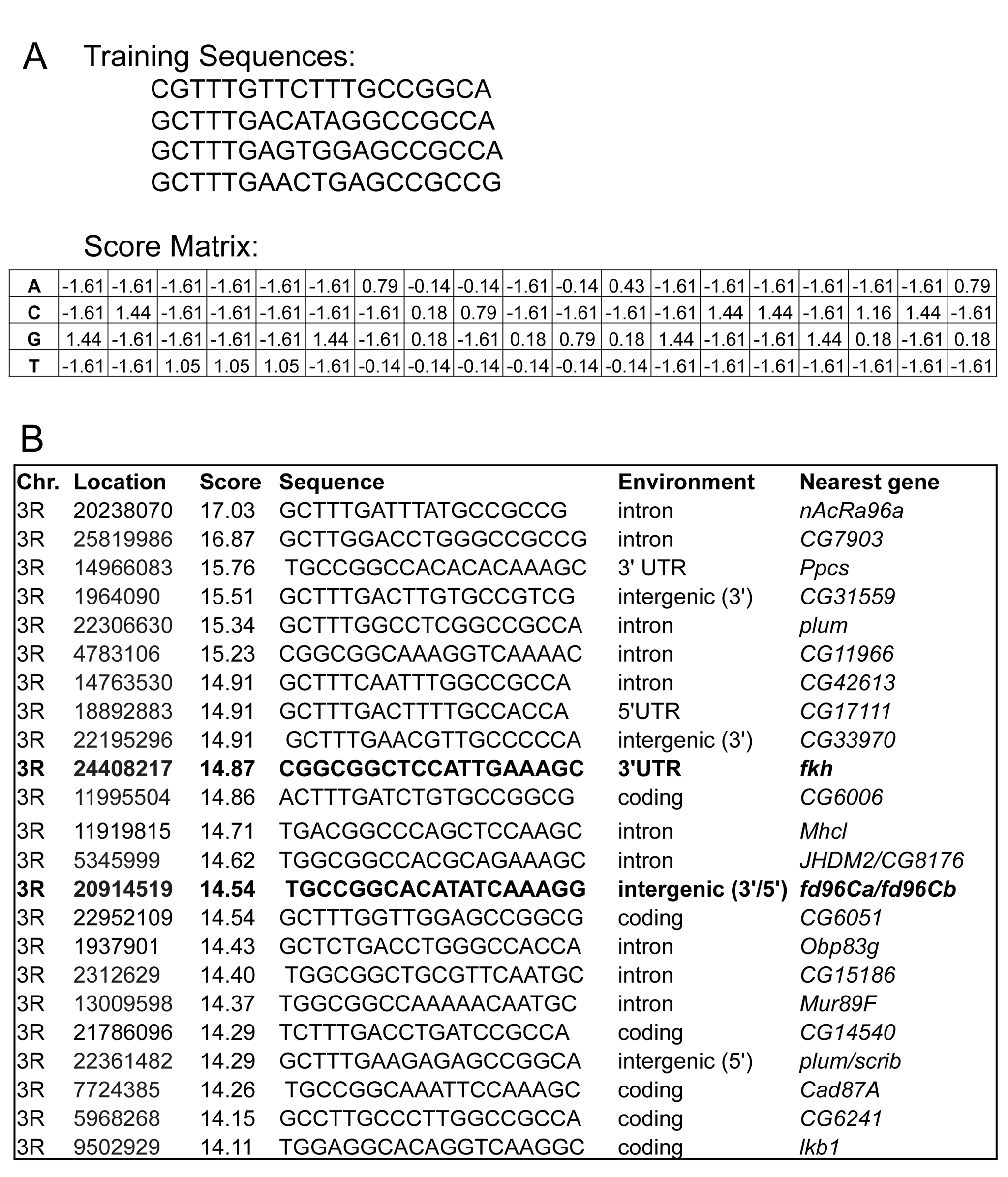

Supplement: Figure S7 — In silico search for FF1 HMG-Helper site pairs. (A) Training sequences and PWM used in a search of Chromosome arm 3R. (B) List of the top 23 hits, location and genomic environment. (TIF) [file pgen.1004591.s007.tif]

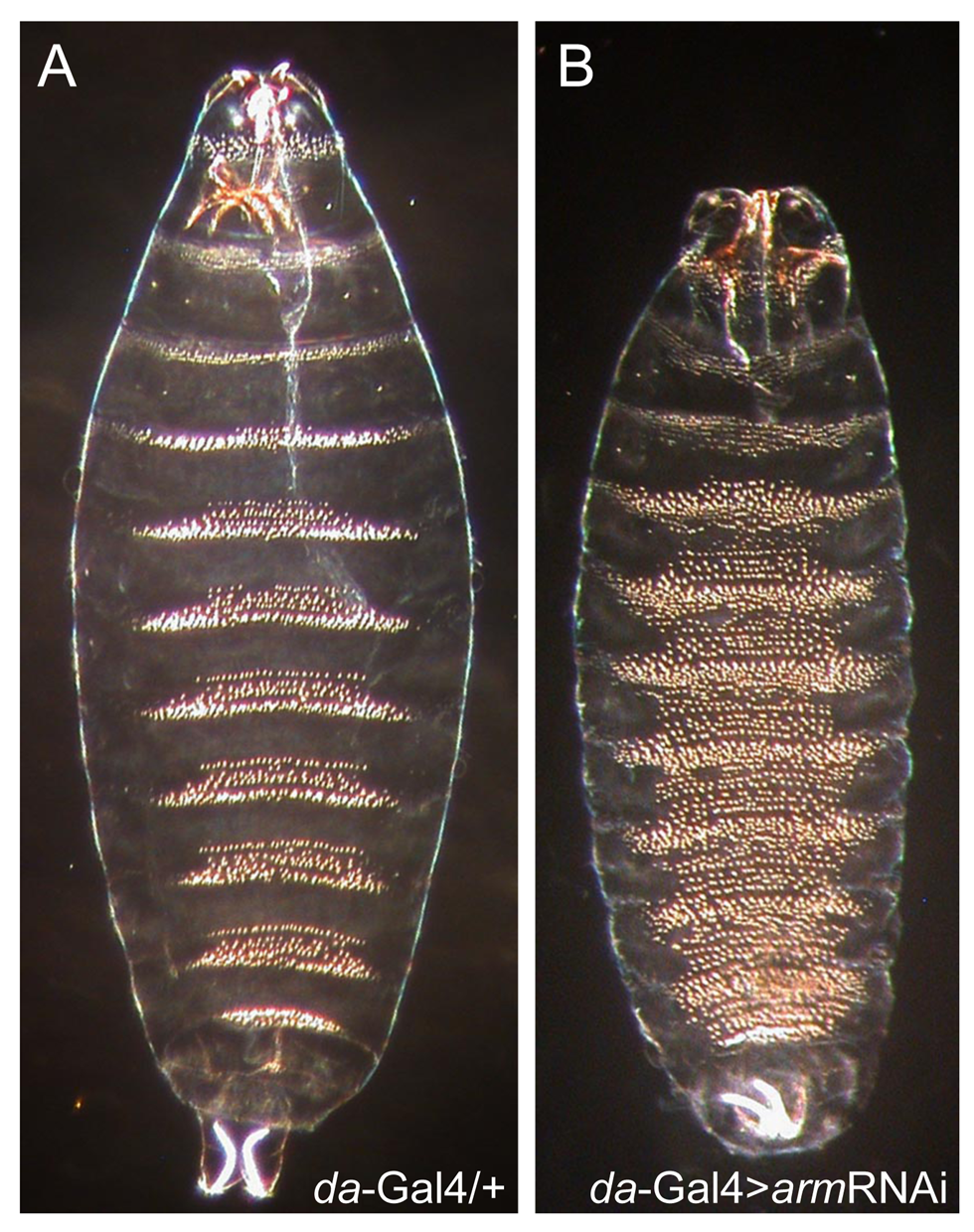

Supplement: Figure S8 — Da-Gal4>UAS-ArmRNAi embryos secrete cuticle with hallmarks of reduced Wg signaling. Darkfield micrographs of end stage embryo cuticles from animals containing a P[da-Gal4] transgene and control (A) or P[UAS-armRNAi] chromosome (B). The Arm depleted embryos have extra denticles indicative of a partial loss of Wg signaling [63], [71], but lack the gross abnormalities associated with a loss of cell adhesion [69], [70]. (TIF) [file pgen.1004591.s008.tif]

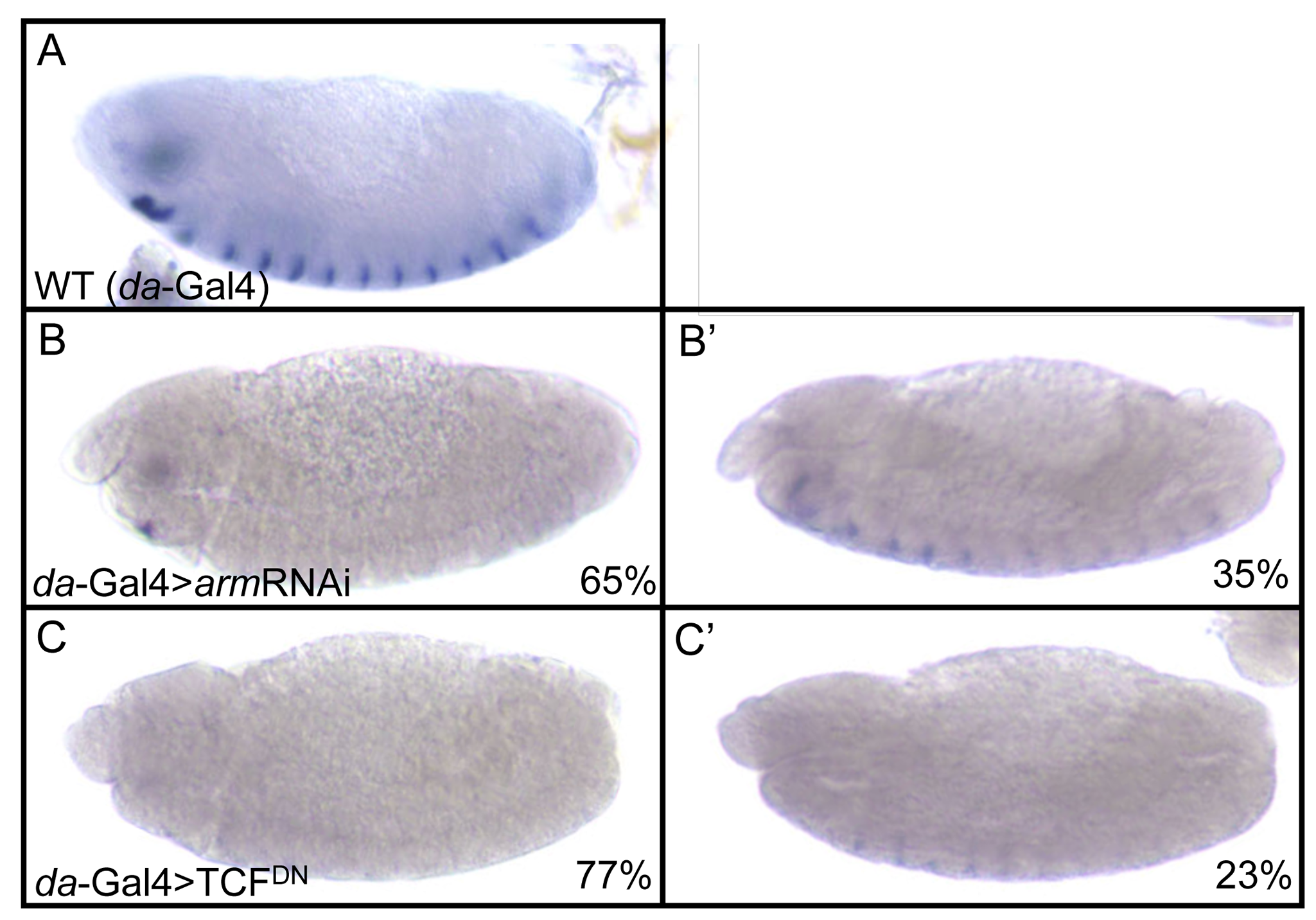

Supplement: Figure S9 — fd96Cb is activated by Wg signaling in the embryo. Bright field images of stage 13 embryos with in situ hybridization using dioxigenin-labeled probe complementary to the fd96Cb transcript. (A) Control embryos exhibited ventral stripes, similar to the fd96C W-CRM reporter. This expression was lost (B,C) or severely reduced (B′,C′) in embryos where armRNAi (B,B′) or TCFDN (C,C′) were ubiquitously expressed via the da-gal4 driver. The percentage of embryos displaying reduced or complete loss of signal is indicated in the bottom right corner of each panel (armRNAi, n = 20; TCFDN n = 52 embryos). (TIF) [file pgen.1004591.s009.tif]

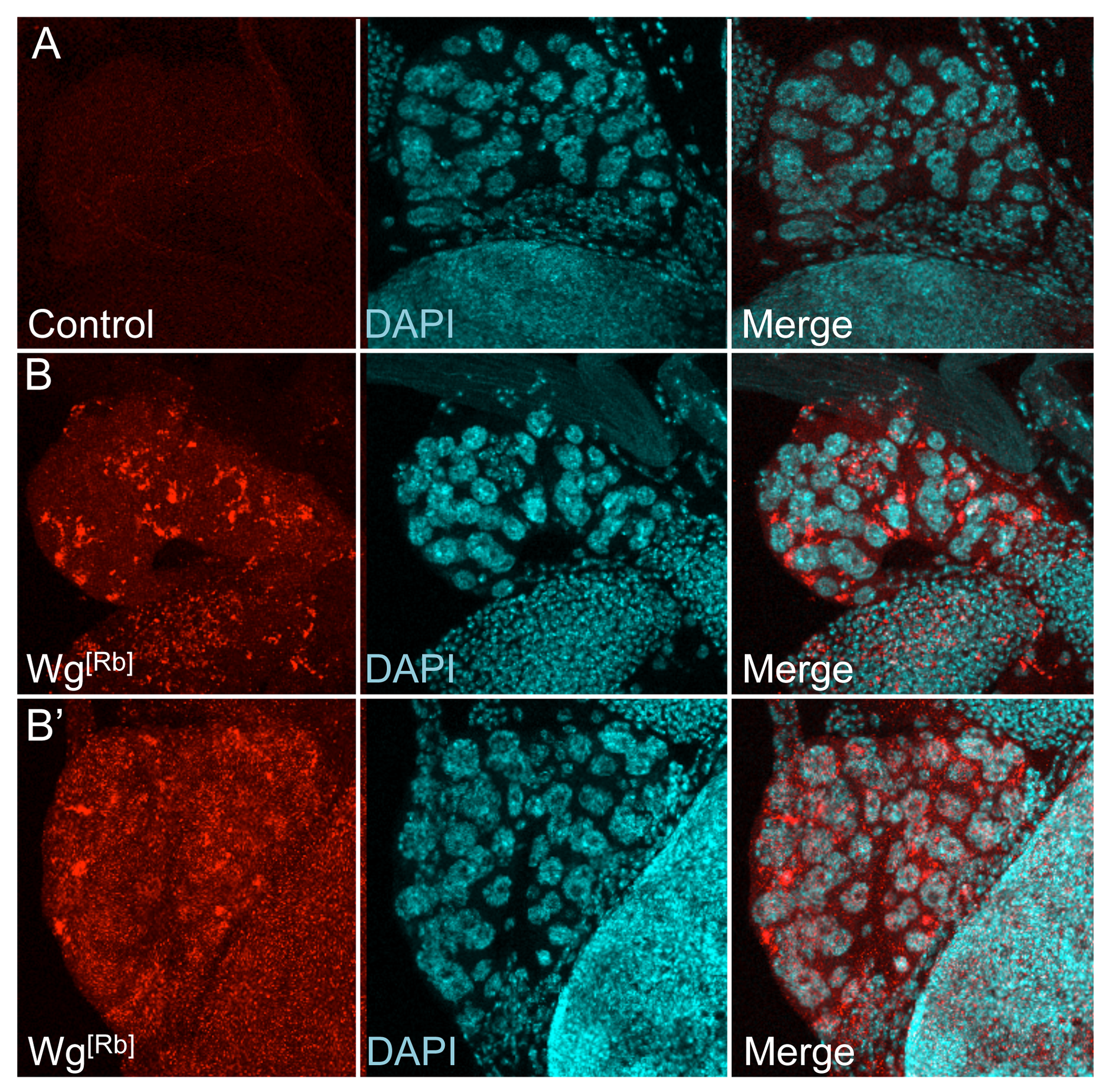

Supplement: Figure S10 — Wg expression in the ring gland using an affinity purified rabbit anti-Wg antisera. (B–B′) Confocal images of Wg immunostains (red) and DAPI (blue) demonstrating Wg expression in the ring gland (bottom two rows). Omitting the 1° antibody results in no signal (A). (TIF) [file pgen.1004591.s010.tif]
